# Supplementary figures and images for: Detecting behavioural changes in human movement to inform the spatial scale of interventions against COVID-19
Source: PLoS Comput Biol. 2021 Jul 12;17(7):e1009162. doi: 10.1371/journal.pcbi.1009162 (PMC8297940; doi:10.1371/journal.pcbi.1009162)

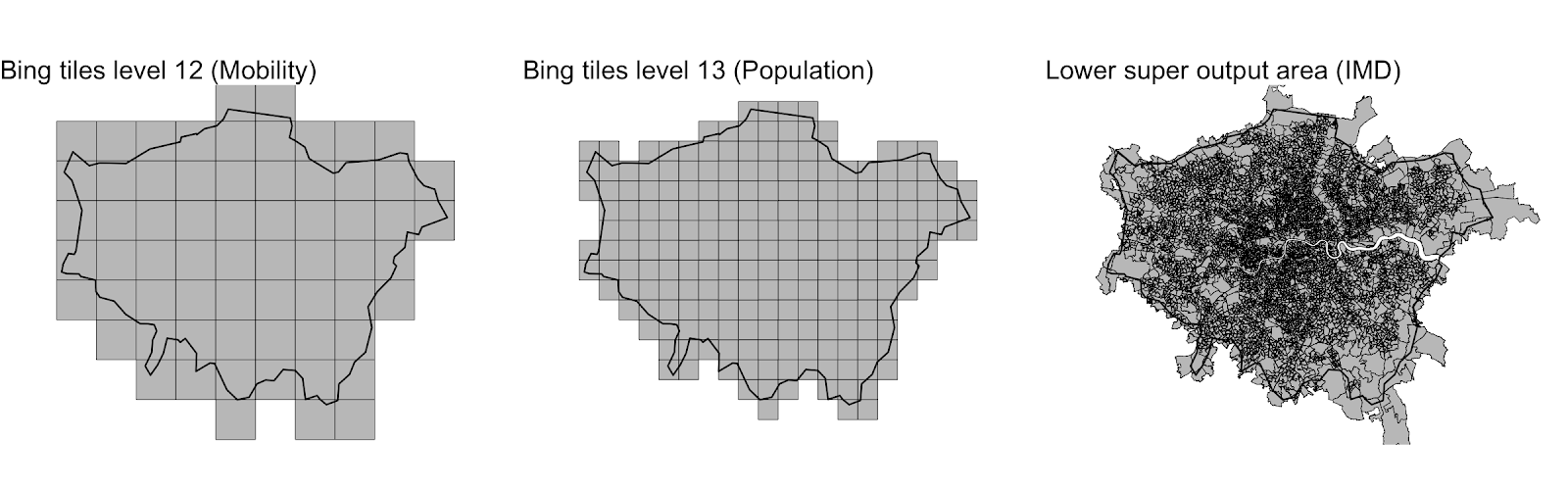

Supplement: S1 Fig — a) Zoom level 12 tiles, b) Zoom level 13 tiles, c) Lower Super Output Areas. Base map data from Natural Earth and Office for National Statistics [55–56]. (TIF) [file pcbi.1009162.s001.tif]

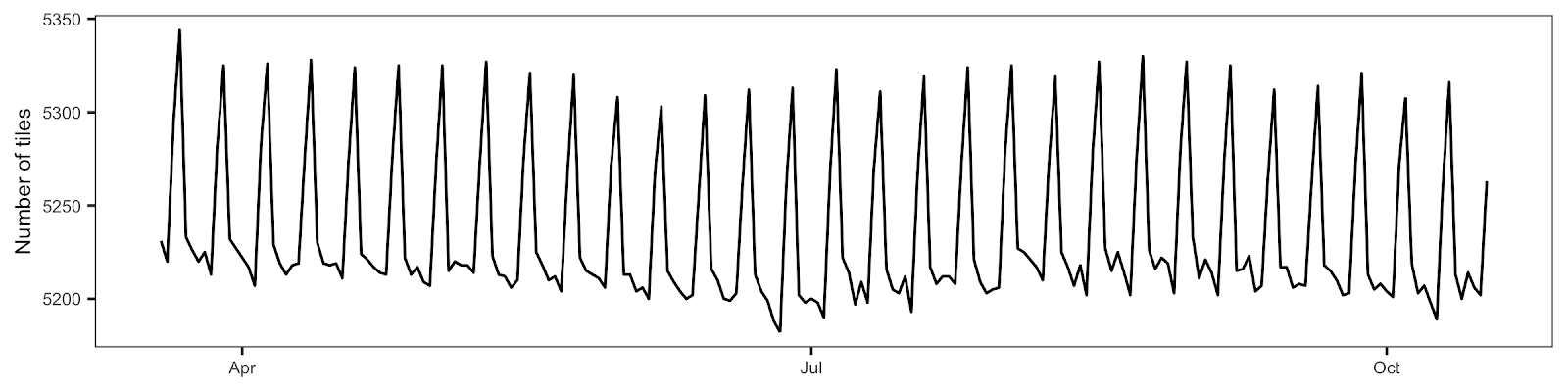

Supplement: S2 Fig — Cells recording fewer than 10 persons moving between cells along any connection are censored from the dataset to preserve user privacy. (TIF) [file pcbi.1009162.s002.tif]

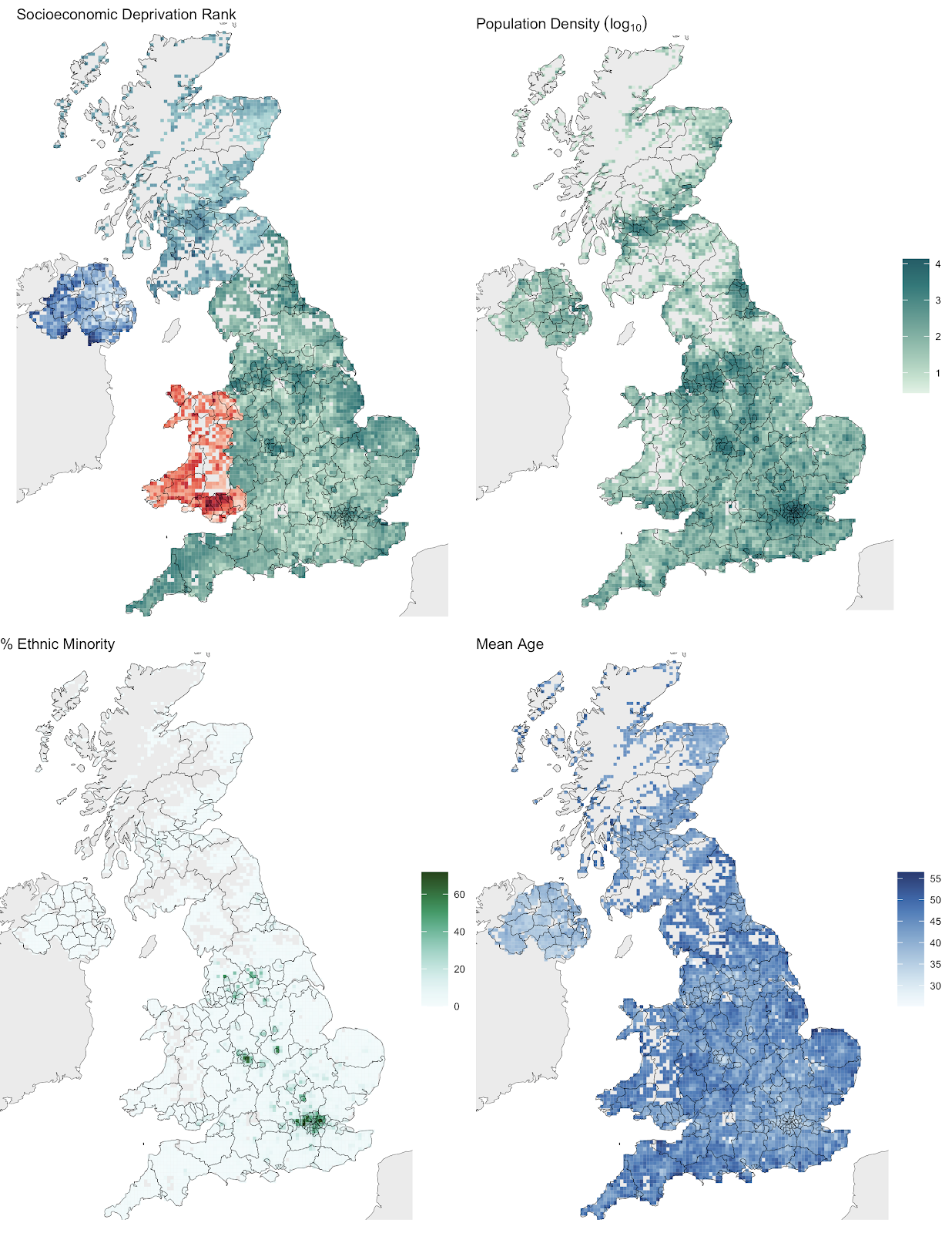

Supplement: S3 Fig — In each case, a white cell means the data were missing from the Facebook mobility data and so are not displayed here. In most cases this is due to censoring of low numbers, except for the small discontinuity around Swindon, mentioned in the Main Text. a) IMD rank. Each country has a different colour because the measure of IMD is different in each country. In each case, the darker shade is higher IMD. b) Population density per cell (log scale). c) Percentage of the population self-identifying as any other ethnicity than “Any white background”. d) Mean age of the population resident in each cell. Base map data from Natural Earth [55]. (TIF) [file pcbi.1009162.s003.tif]

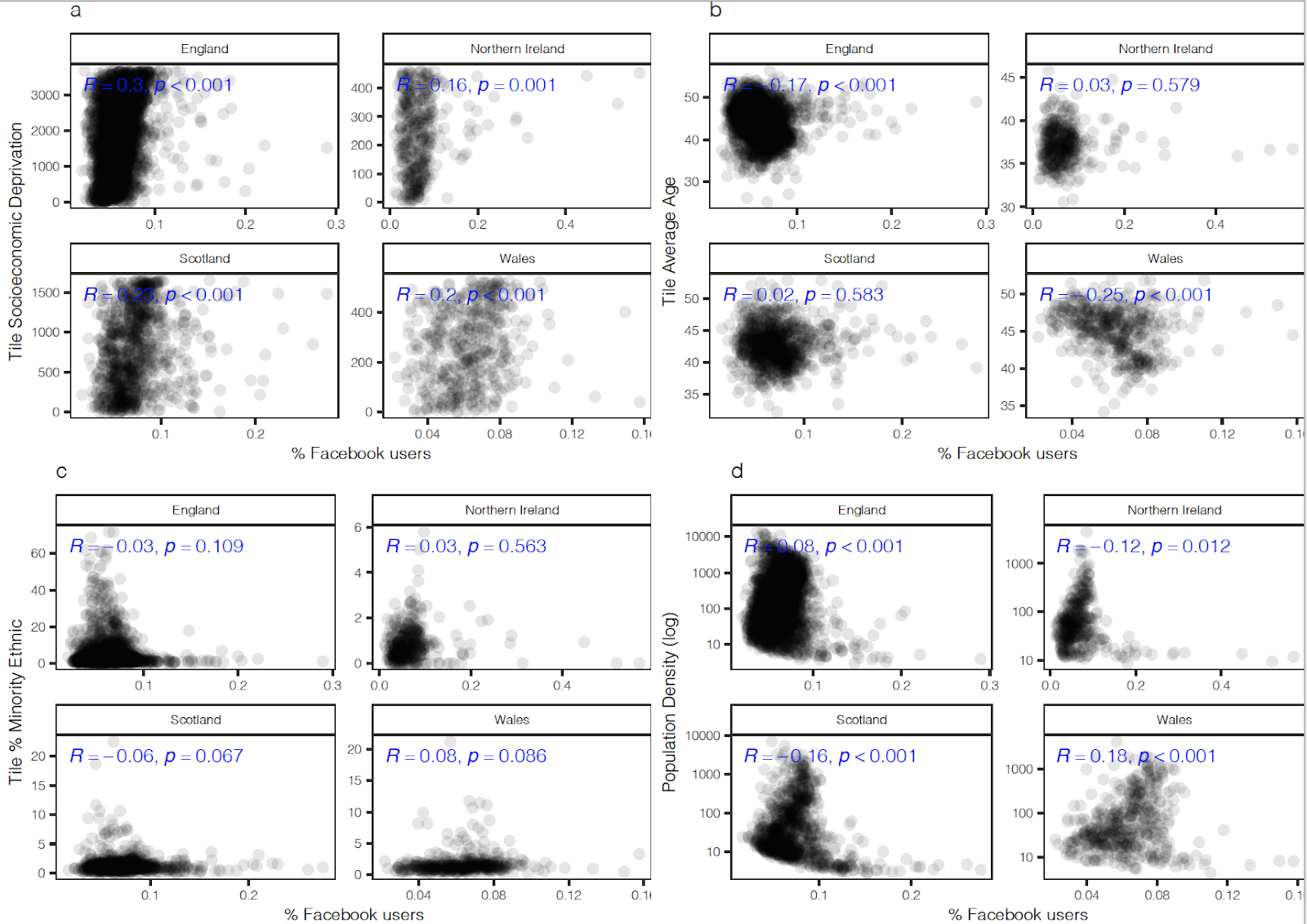

Supplement: S4 Fig — a) IMD, b) mean age, c) percent minority ethnic, and d) population. Variables were aggregated from mid-level census geographies for each country. The mean value of each variable was assigned to intersecting tiles, weighted by small area population estimates. Correlation is shown on the panel as the Pearson correlation coefficient (R) and two-sided p values. (TIF) [file pcbi.1009162.s004.tif]

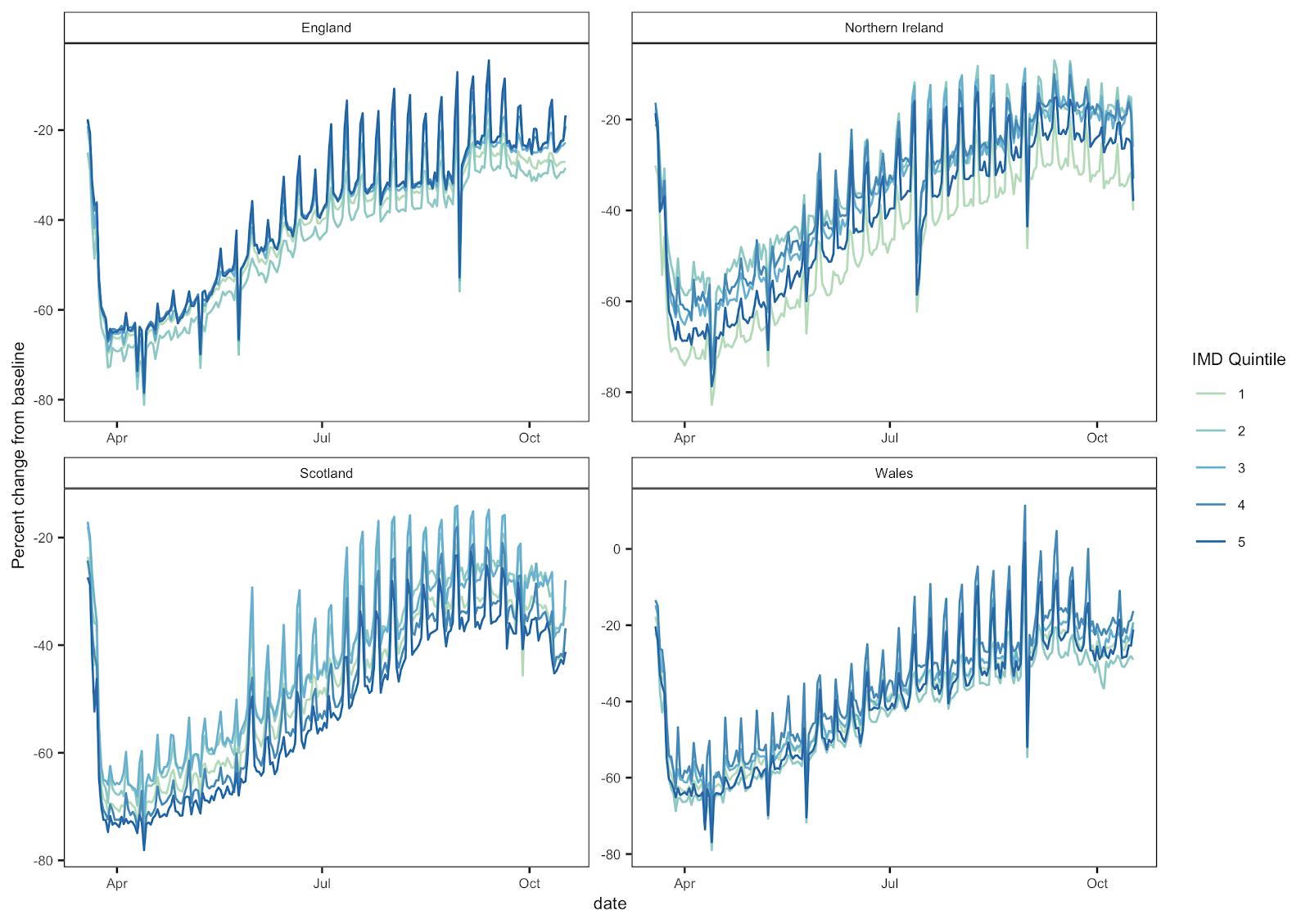

Supplement: S5 Fig — Percent change from baseline for movement between cells by IMD quintiles in each country. IMD data was aggregated to cell level and weighted by small area population estimates. IMD quintiles range from 1 (most deprived) to 5 (least deprived). (TIF) [file pcbi.1009162.s005.tif]

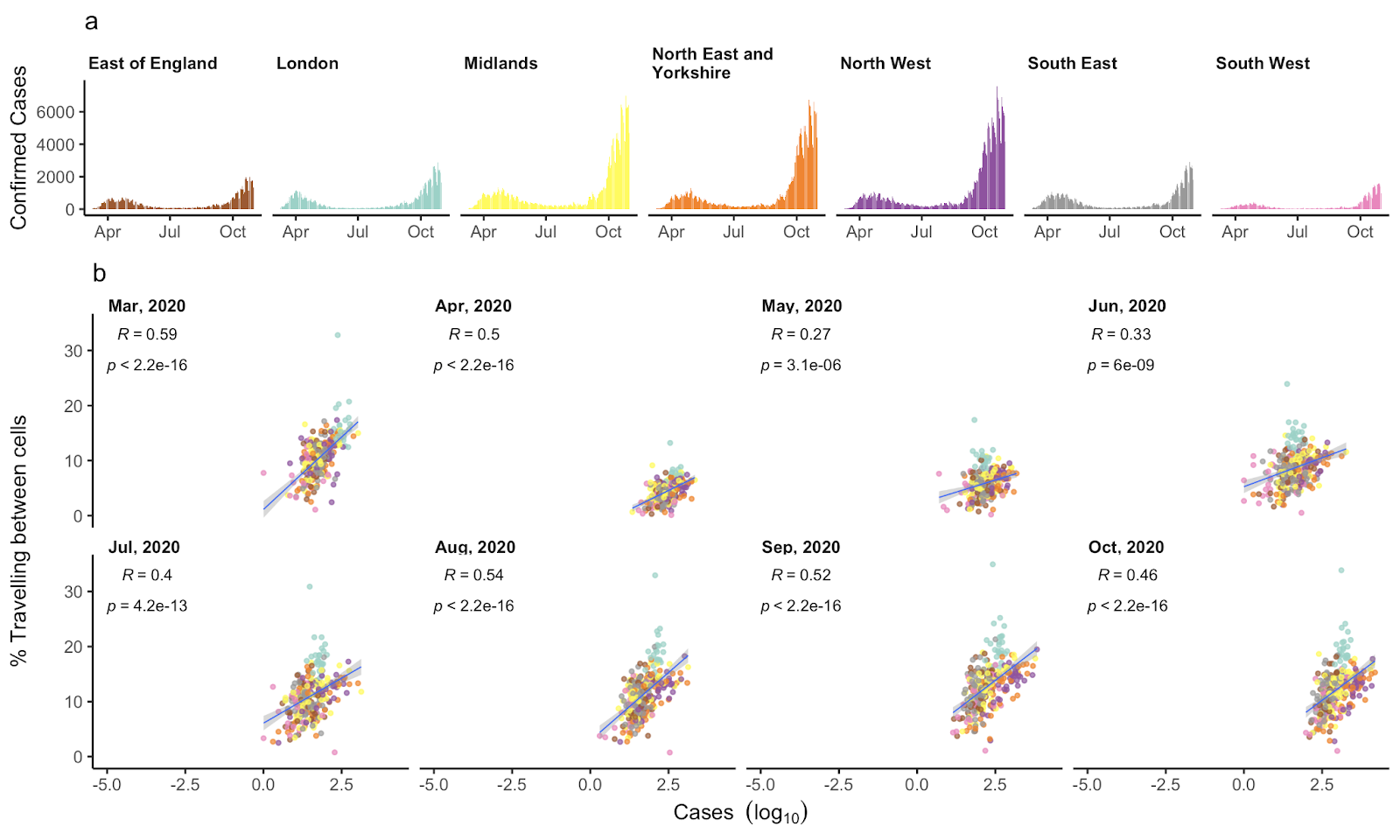

Supplement: S6 Fig — a) Daily confirmed SARS-CoV-2 tests in each NHS region. b) The relationship between the percentage of users travelling outside their cell and the total number of confirmed SARS-CoV-2 positive tests, by month of the study period. Dots show lower-tier local-authorities coloured by their NHS region as in panel a. (TIF) [file pcbi.1009162.s006.tif]

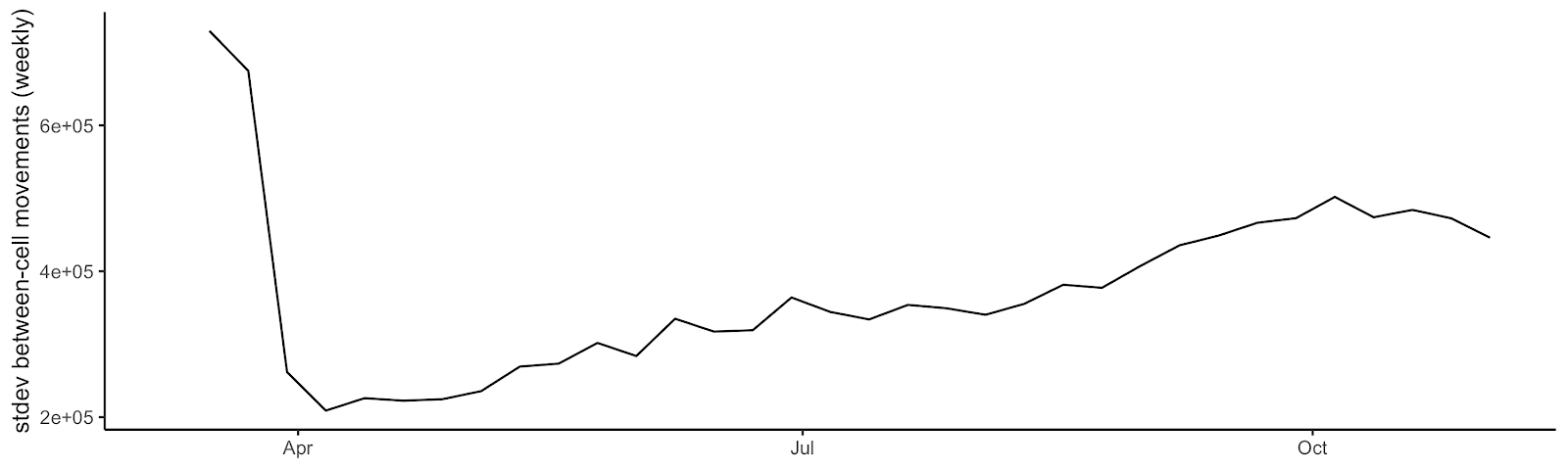

Supplement: S7 Fig — The standard deviation of between cell movements through time. Decreased variance indicated smaller differences in daily between-cell travel measurements per week. This reflects a reduction in the weekly pattern of between-cell movements. (TIF) [file pcbi.1009162.s007.tif]

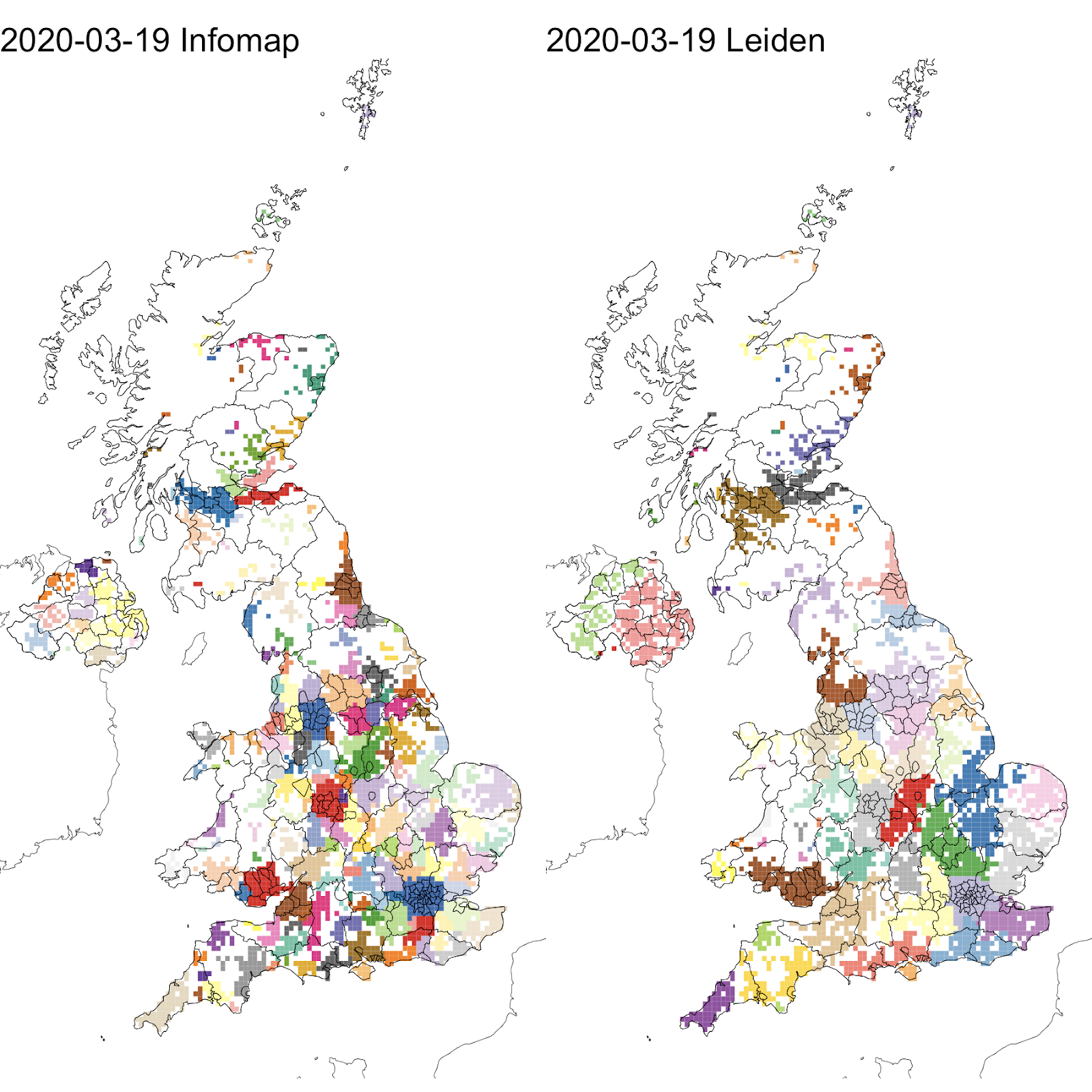

Supplement: S8 Fig — The extent of communities detected by InfoMap (a) and Leiden (b) on March 19th. Leiden communities are largely a superset of communities detected by Infomap, indicating the detection of a different hierarchical structure, but an agreement of community boundaries between the two algorithms. Base map data from Natural Earth [55]. (TIF) [file pcbi.1009162.s008.tif]

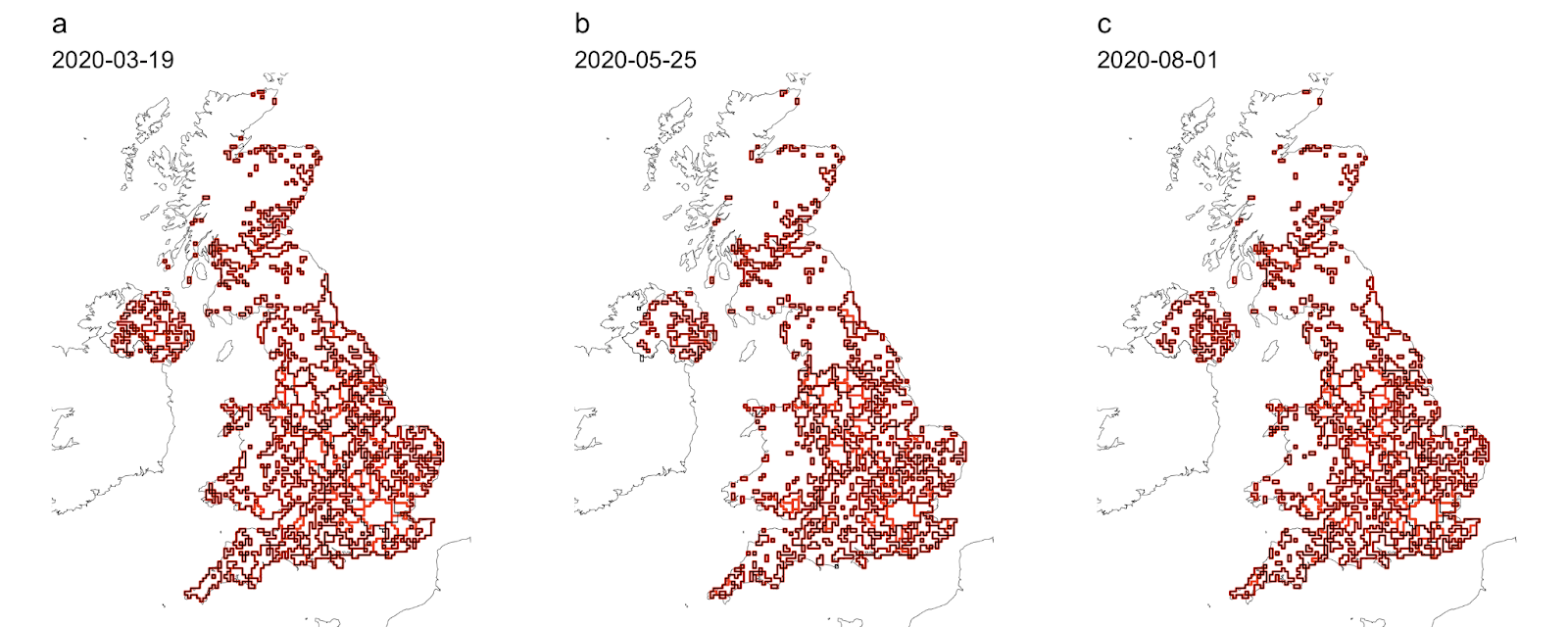

Supplement: S9 Fig — A comparison of communities detected with the InfoMap (red) and Leiden (black) methods for a selection of dates. Base map data from Natural Earth [55]. (TIF) [file pcbi.1009162.s009.tif]

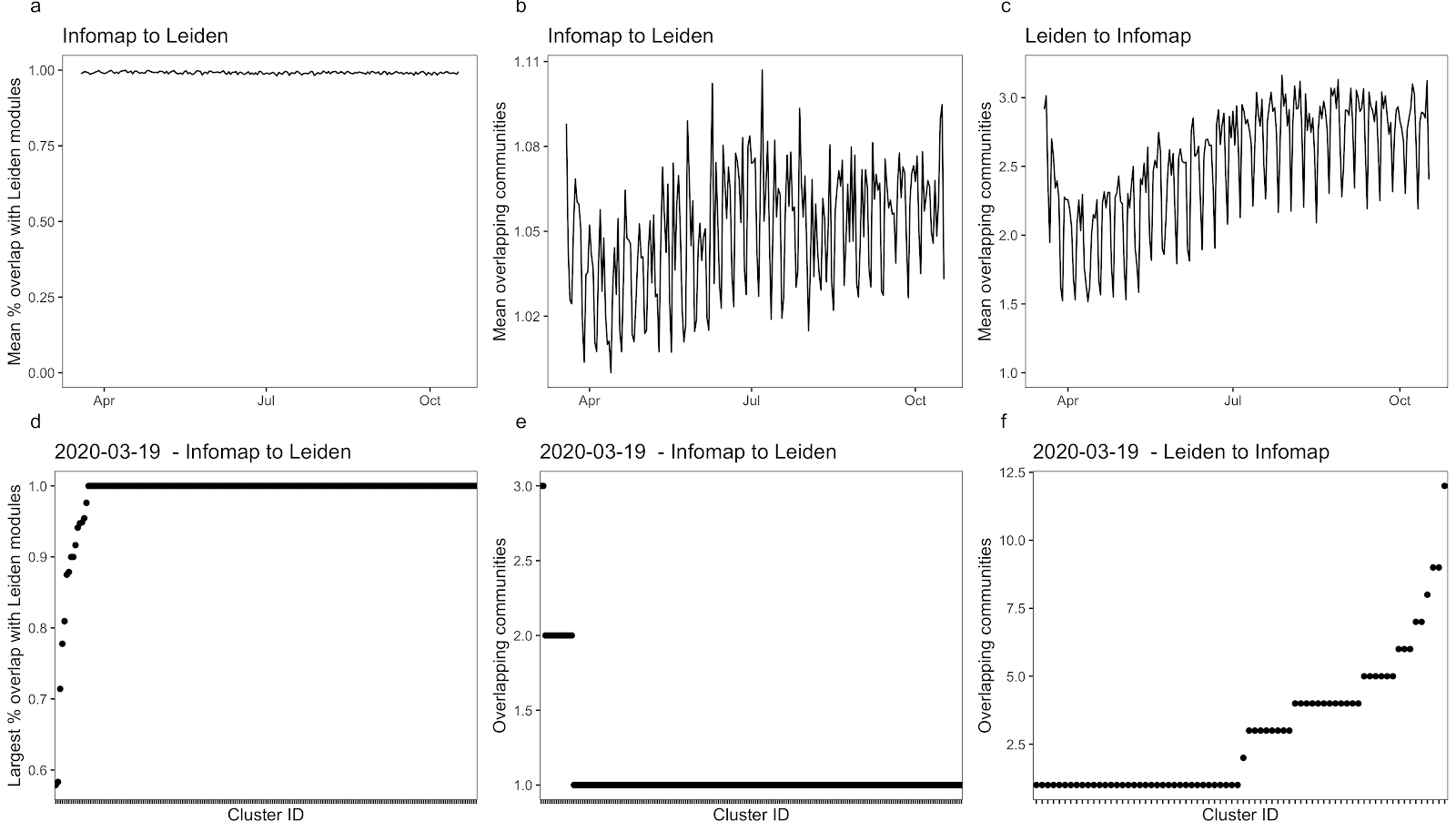

Supplement: S10 Fig — A comparison of the spatial intersection between communities detected by the Infomap and Leiden algorithms through time. a) The average % overlap of InfoMap communities with Leiden communities. b) The number of Leiden communities each InfoMap community intersects, and c) the inverse comparison of Leiden communities to InfoMap communities. For a specific date, d) the maximum % areal overlap of each InfoMap community with all Leiden communities. e) The number of Leiden communities each InfoMap community intersects, and c) the inverse comparison of Leiden communities to InfoMap communities. This shows spatial alignment between InfoMap and Leiden communities, where Leiden communities tend to be larger than those detected by InfoMap. (TIF) [file pcbi.1009162.s010.tif]

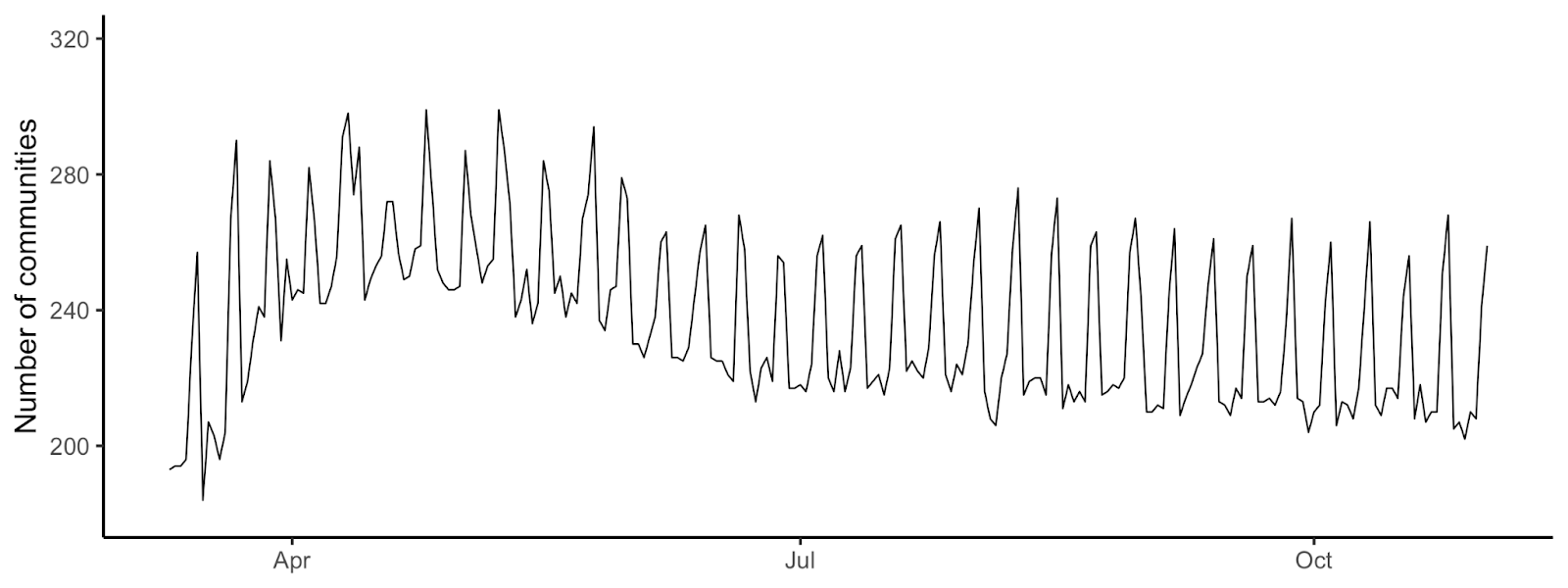

Supplement: S11 Fig — The number (red) of InfoMap communities through time. An increase in the number of communities reflects more local patterns of travel during national interventions. (TIF) [file pcbi.1009162.s011.tif]

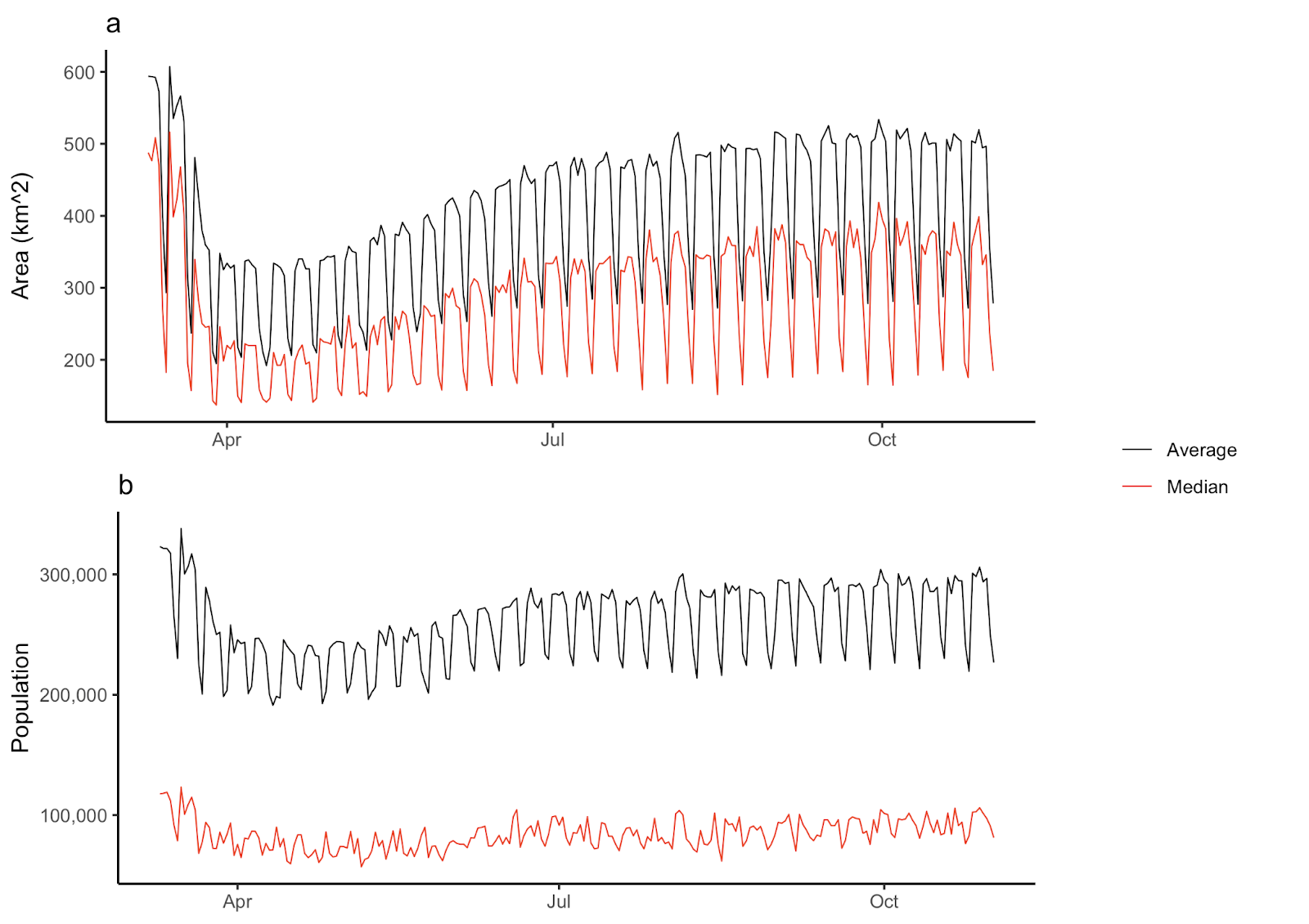

Supplement: S12 Fig — a) The average and median area of InfoMap communities through time. b) The average and median census population of communities through time. The distribution of population is more skewed than that of area, reflecting high populations in specific communities. (TIF) [file pcbi.1009162.s012.tif]

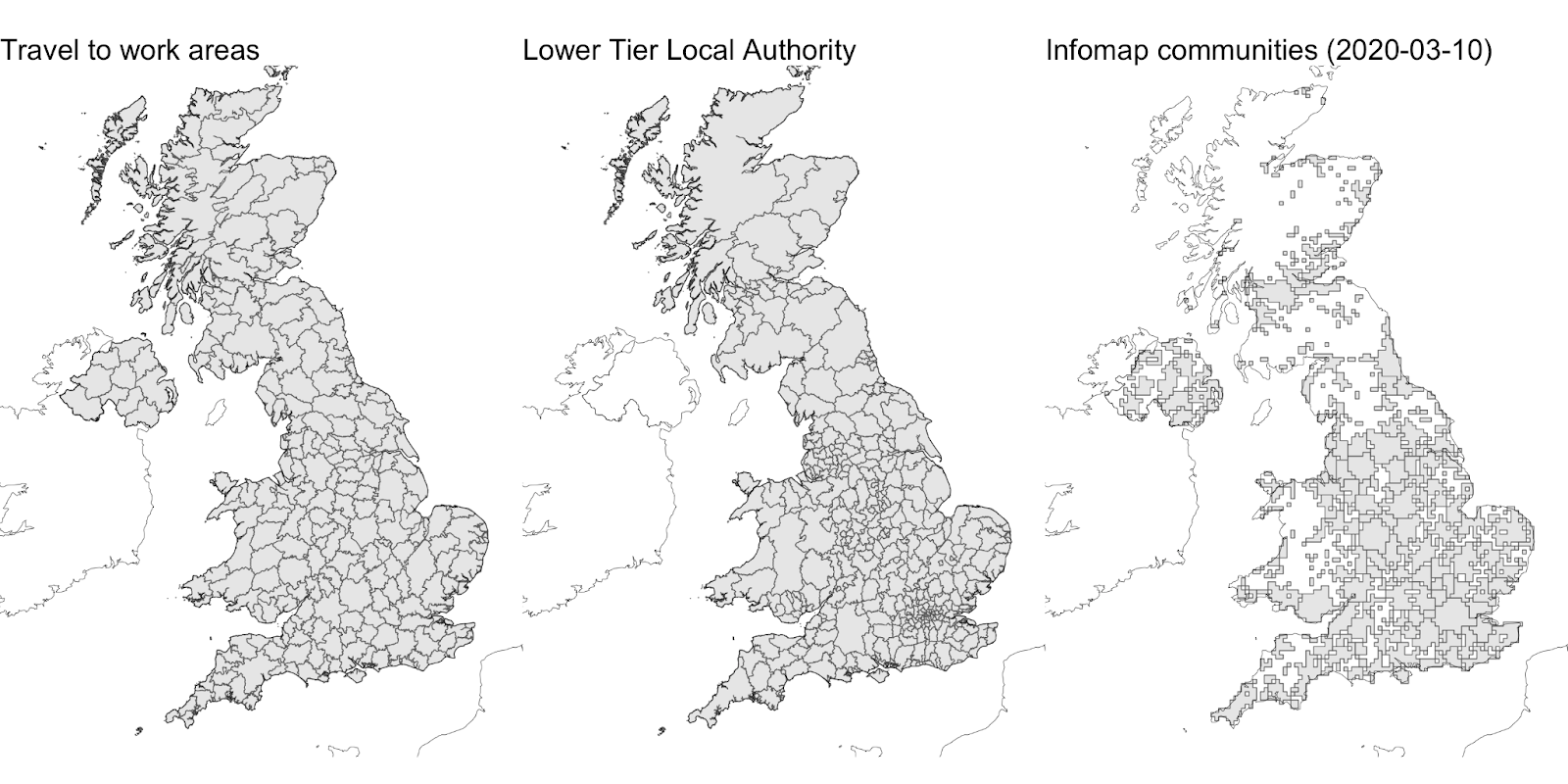

Supplement: S13 Fig — Maps of 2011 TTWAs, LTLAs, and InfoMap communities detected on a specific date. While some InfoMap communities are similar in size to TTWAs and LTLAs, they do not share the same boundaries. There are fewer communities (193) than TTWAs (228) and LTLAs (380) and these communities have a smaller average area across all time periods (389.73 km2) compared to TTWAs (1069.14 km2) and LTLAs (605.28 km2). Base map data from Natural Earth and Office for National Statistics [55,57,58]. (TIF) [file pcbi.1009162.s013.tif]

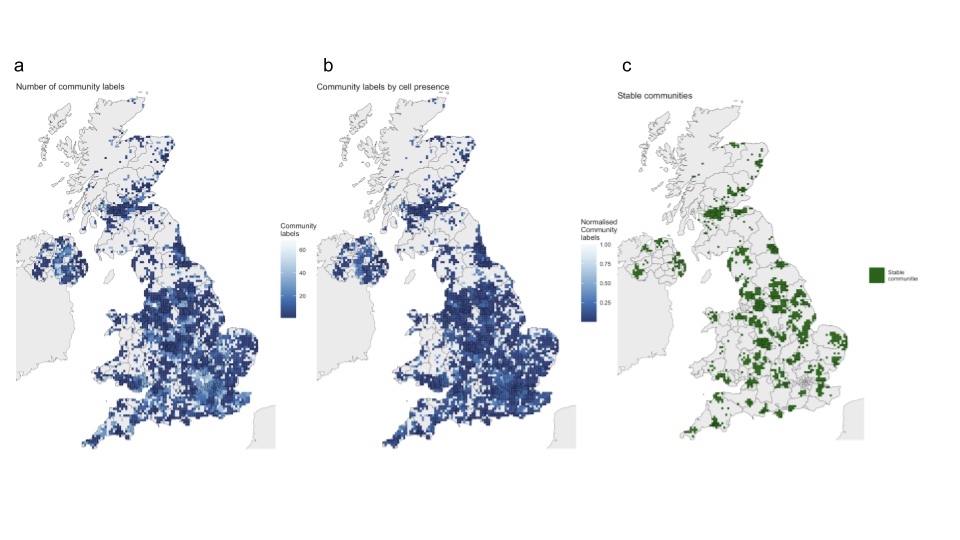

Supplement: S14 Fig — a) the total number of community labels that each cell has had (i.e. number of communities that the cell has ever been in) during the study period. The darkest shade indicates that a cell was always in the same community. b) the number of community labels for a given cell as a proportion of the number of days that cell was present in the dataset. This was calculated as the (number of unique community labels/number of days a cell was present). c) stable communities, marked as those which had the same community label for the entire study period. Base map data from Natural Earth [55]. (TIF) [file pcbi.1009162.s014.tif]

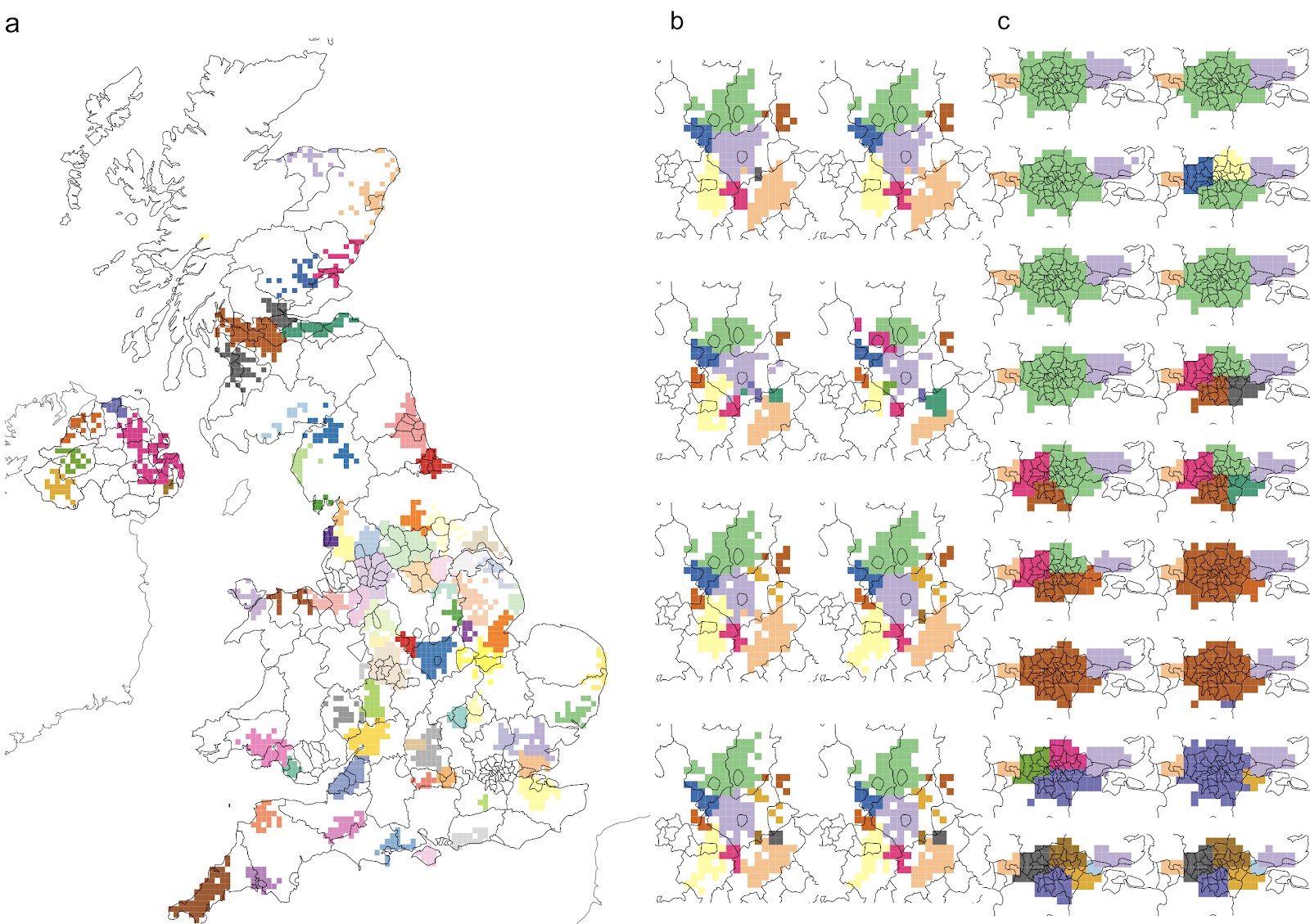

Supplement: S15 Fig — a) The most persistent communities, those that existed throughout the timeseries, as on March 19th, 2020. b) Community membership from March 19th to March 26th, 2020 in Leicestershire, and c) community membership from March 19th to April 5th, 2020 in London. For both figures, panels are ordered row-wise from top left. Base map data from Natural Earth [55]. (TIF) [file pcbi.1009162.s015.tif]

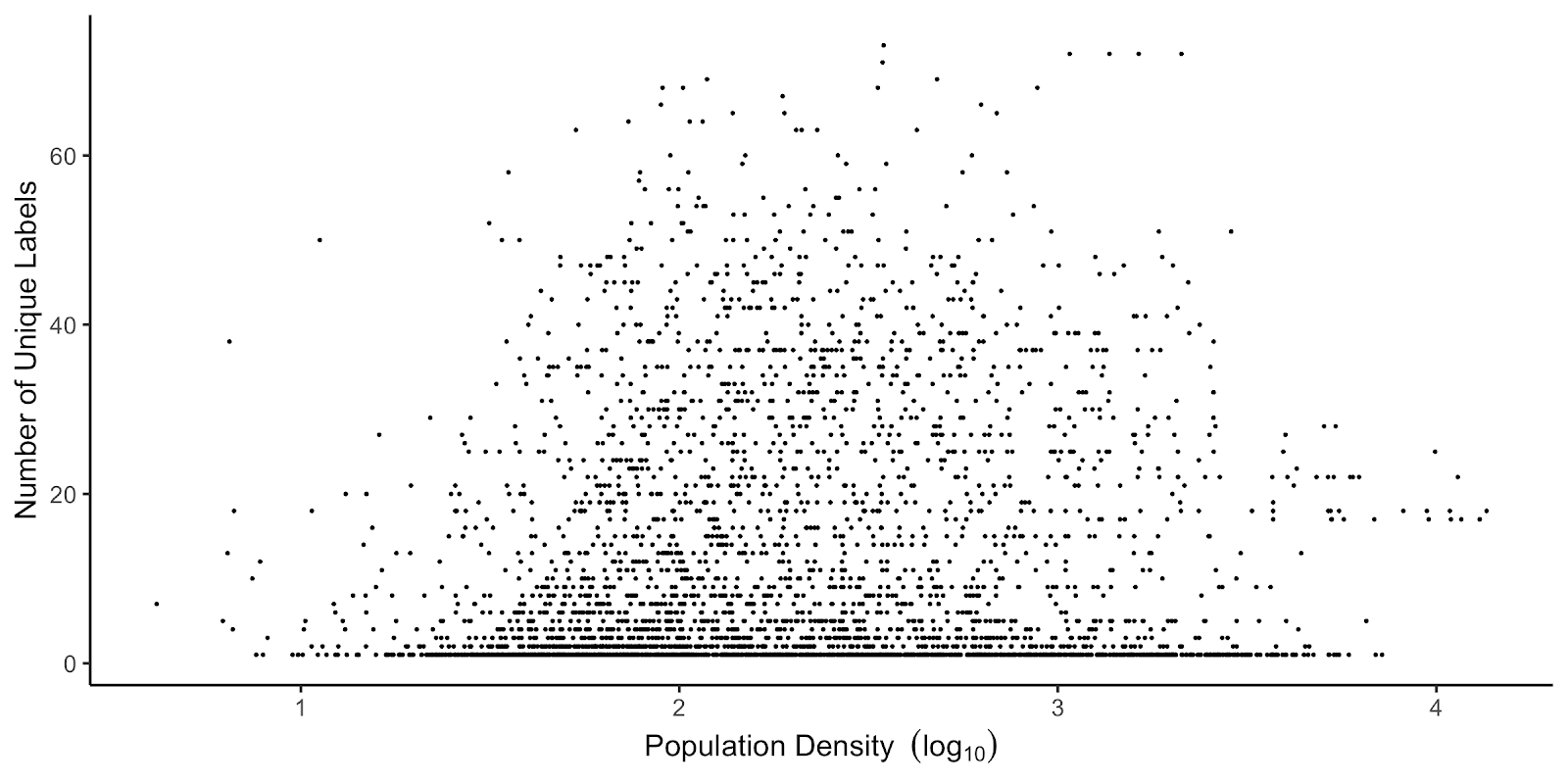

Supplement: S16 Fig — The relationship between the population density of individual cells and the number of community labels assigned during the period. We do not observe a strong association between population density and the number of community labels assigned to individual cells. (TIF) [file pcbi.1009162.s016.tif]

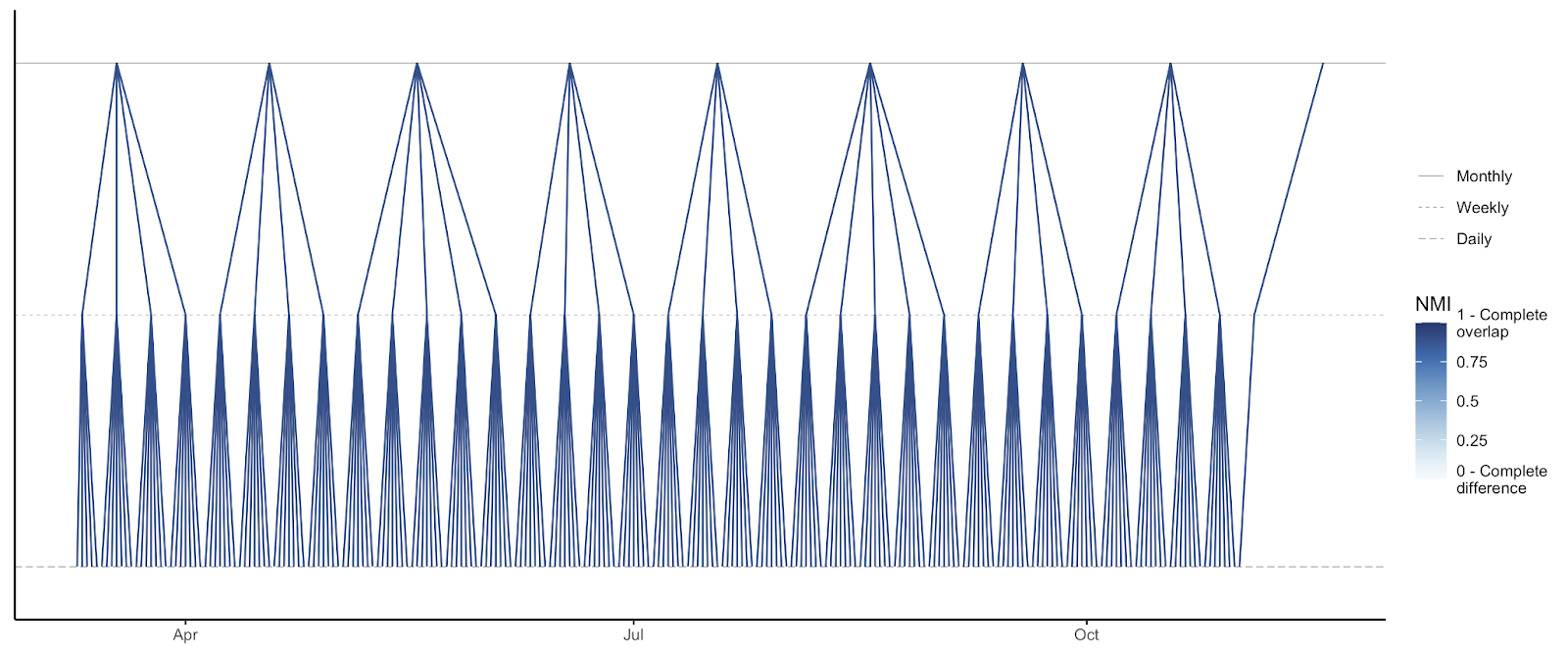

Supplement: S17 Fig — Normalised Mutual Information, a measure of similarity between community partitions, measured for InfoMap communities detected in networks aggregated to daily, weekly, and monthly periods. The partition of each network is compared to its parent time aggregation (days to overlapping weeks, weeks to overlapping months). (TIF) [file pcbi.1009162.s017.tif]

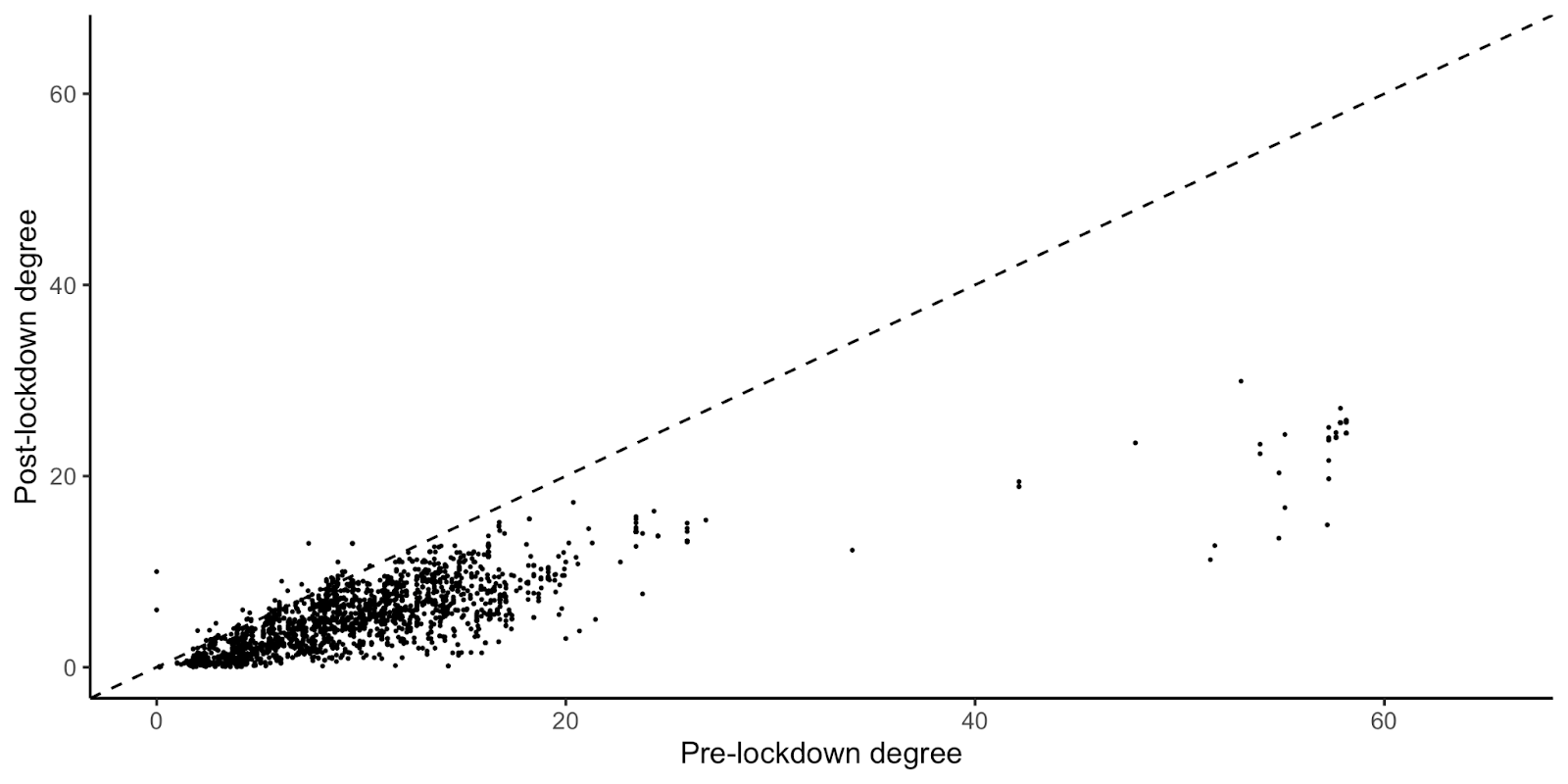

Supplement: S18 Fig — The total degree of movement communities assigned to individual tiles. We observe a relationship between the degree of communities before and during national interventions, indicating that highly connected communities remained highly connected during the period of national interventions. The dashed line indicates where the Pre-lockdown degree is equal to the Post-lockdown degree. All points would fall along this line in the event of no changes to the community network after the intervention. (TIF) [file pcbi.1009162.s018.tif]

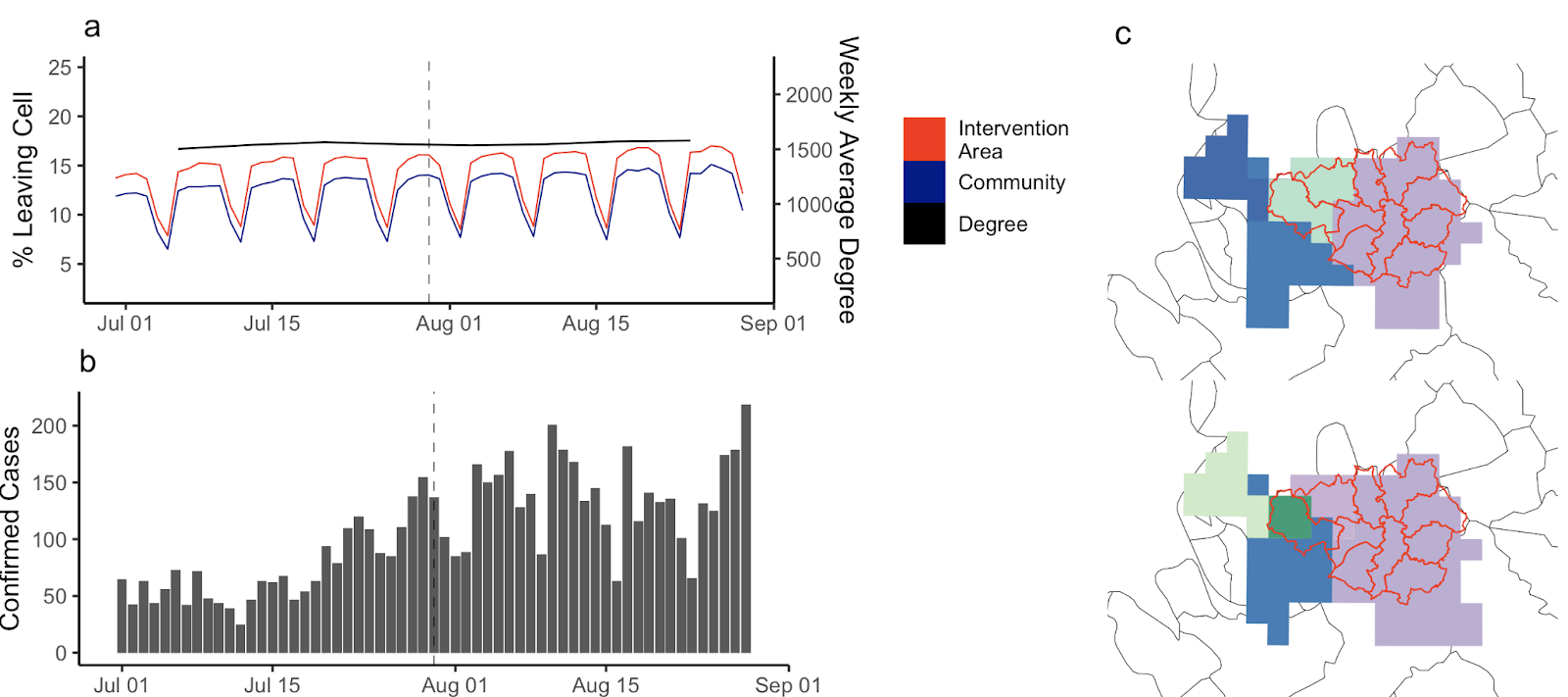

Supplement: S19 Fig — The daily percentage of users travelling between cells in the intervention area and the connected community, and the weekly average degree of intervention cells (a). Confirmed cases in the intervention area (b). Changes in the community structure before and after the introduction of local intervention (c). Base map data from Natural Earth [55]. (TIF) [file pcbi.1009162.s019.tif]

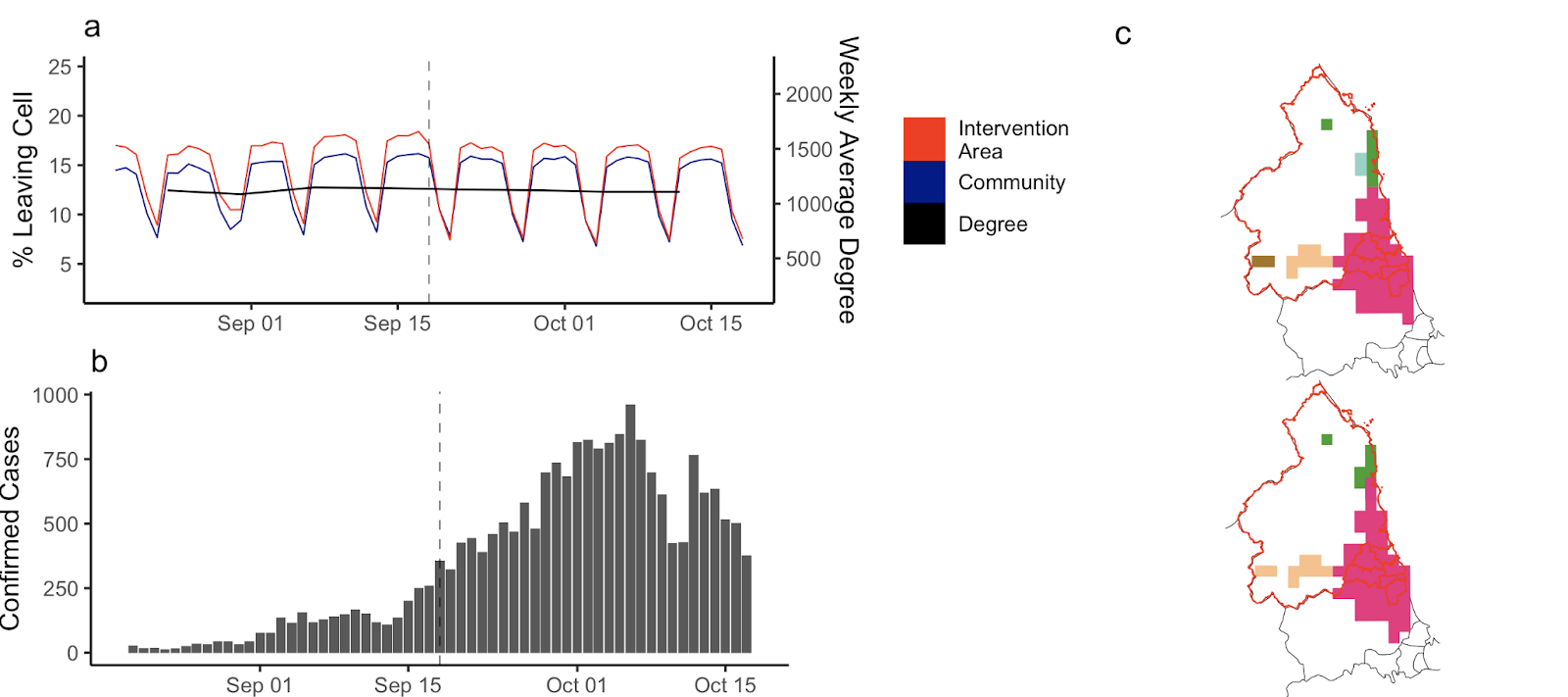

Supplement: S20 Fig — The daily percentage of users travelling between cells in the intervention area and the connected community, and the weekly average degree of intervention cells (a). Confirmed cases in the intervention area (b). Changes in the community structure before and after the introduction of local intervention (c). Base map data from Natural Earth [55]. (TIF) [file pcbi.1009162.s020.tif]

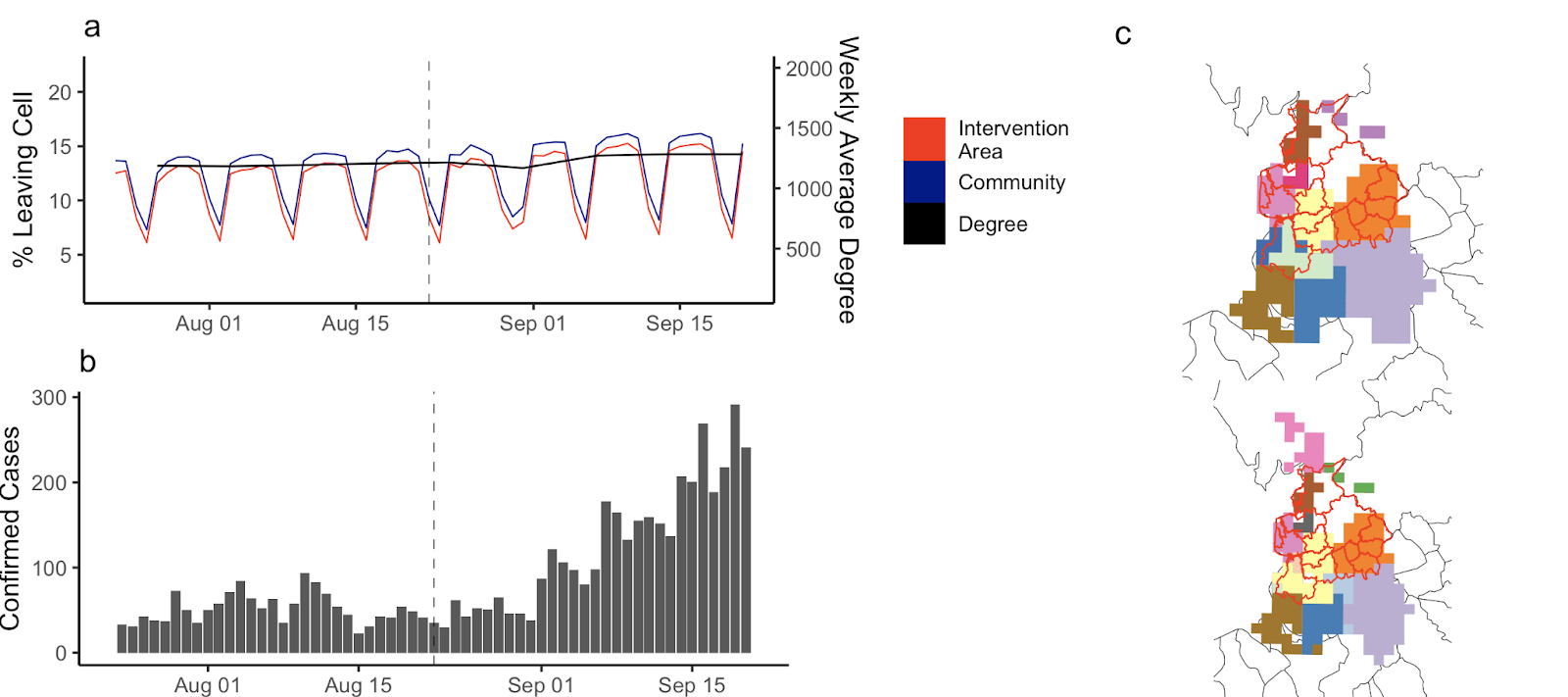

Supplement: S21 Fig — The daily percentage of users travelling between cells in the intervention area and the connected community, and the weekly average degree of intervention cells (a). Confirmed cases in the intervention area (b). Changes in the community structure before and after the introduction of local intervention (c). Base map data from Natural Earth [55]. (TIF) [file pcbi.1009162.s021.tif]
