# Supplementary material for: Detecting behavioural changes in human movement to inform the spatial scale of interventions against COVID-19
Source: PLoS Comput Biol. 2021 Jul 12;17(7):e1009162. doi: 10.1371/journal.pcbi.1009162 (PMC8297940; doi:10.1371/journal.pcbi.1009162)
Supplement: Attachment — Submitted filename: [Review 1] UK Movement Communities.docx [file pcbi.1009162.s022.docx]

**Detecting behavioural changes in human movement to inform the spatial scale of interventions against COVID-19.**

## Dear Editor,

## Thank you for the opportunity to revise our paper for PLOS Computational Biology. We have made the changes requested by the reviewers, clarified the motivation of the paper as well as the findings, and conducted the extra analyses requested. This has improved the quality of the paper and we thank the reviewers for their efforts and their useful suggestions.

## We hope by addressing all of the reviewer comments thoroughly, the paper will be suitable for publication in PLOS Computational Biology.

## Best wishes,

## Hamish Gibbs

# Reviewer 1

## Reviewer 1 - Summary

In this work, the authors use human mobility data from Facebook users in the UK to analyze the associated spatial network of movements before, during and after the national lockdown and in the period of local interventions. They applied community detection techniques and measured the evolution of the detected communities over time. These analyses revealed that the mobility network became sparser, the number of communities was decreased, and long-distance journeys were less frequent during the lockdown. Local interventions did not change the community structure and only decreased travel outside of local areas weakly.

The authors address a highly relevant topic with potentially big implication for public health and future implementations of non-pharmaceutical interventions against the spread of infections diseases. The authors make sure that their data set is not biased by comparing the local fraction of Facebook users between regions and checking for correlations with census variables.

However, I have reservations regarding the interpretation, implications, and novelties of this study. The paper lacks a clear story and does not live up to its title, because I cannot identify the “spatial scale of interventions against COVID-19”.

We thank the reviewer for these positive comments. In response, we have made changes to clarify the overall message and main results of the paper. We have adopted the reviewer’s suggestions of citations supporting the connection between movement and disease transmission, and have updated the results section with a more detailed analysis of the changes in network and community structure of the UK movement network through time. The changes suggested by all the reviewers have helped to support the “spatial scale” focus of the article.

## Reviewer 1 - Major 1

The correlation between movement and new COVID-19 cases was reported in many previous studies, eg in Badr, Hamada S., et al. "Association between mobility patterns and COVID-19 transmission in the USA: a mathematical modelling study." The Lancet Infectious Diseases 20.11 (2020): 1247-1254. This should be at least mentioned, if not discussed.

We agree that this is an important relationship and thank the reviewer for suggesting this reference. We have included a more thorough discussion of the relationship between movement and COVID-19 cases in the introduction:

The spread of COVID-19 through travel networks has been demonstrated in China, where connectivity to Wuhan was shown to predict the timing of arrival of COVID-19 cases in each region (11,12). The volume of mobility has also been shown to correlate with transmission of COVID-19, where movement can be used as a proxy for the measurement of the degree of social distancing [(13)](https://www.zotero.org/google-docs/?broken=SfVdZs). Travel and movement behavior during epidemics may also change in response to imposed interventions, perceived risk, and due to seasonal activities such as vacations [(14,15)](https://www.zotero.org/google-docs/?broken=xvkMIw). During the COVID-19 pandemic, mobility data has been used to assess adherence to movement restrictions (16,17), the impact of movement restrictions on the transmission dynamics of COVID-19 (11,18,19), and the socioeconomic impacts of large scale movement restrictions (20,21).

And in the discussion section:

There is a demonstrated relationship between human mobility and transmission of COVID-19 relating both to the spatial introduction of disease, where connectivity is correlated with the time of first introduction (11), and rates of movement in specific locations, which are correlated with disease transmission (13).

## Reviewer 1 - Major 2

To quantify changes in the network structure the authors calculate the edge betweenness of connections. It is unclear whether the authors assigned weights to the links when computing this quantity. If not, it seems to me that only the removal of links due to the censoring of links with less than 10 travelers changes the observable. Also, the motivation for this observable is not clear to me. What do we learn about behavioural changes from it, additionally to just quantifying the length and frequency of trips?

We have added the clarification to the text that edge betweenness was calculated on the weighted directed network. We have modified the methods by adding a section on the calculation of edge betweenness centrality:

### **Edge betweenness centrality**

We computed the edge betweenness centrality of the movement network, a measure of which edges are most responsible for maintaining connection between different parts of the network. Betweenness centrality was calculated for the weighted, directed network. Comparing this measure of centrality provides information about changes in network topology, the volume of travel along the network, and which connections might be preferred targets for interventions.

And also added this information where the results are discussed:

We observe a disproportionate reduction in journeys which were highly central before the introduction of national restrictions, reflecting the reduction of travel along long distance connections (Figure 3 e, f) which tend to be highly central in the network, and an increased fragmentation of the network.

Because edge betweenness is calculated on the weighted network, it is not only the removal of links due to censoring, but also the decrease or change in weight that affects the value. The change in which links have the highest edge betweenness provides information both about changes in the network topology and variations in the volume of travel along specific edges in the network.

## Reviewer 1 - Major 3

Next, in the central part of the paper, the authors identify communities in the network and, subsequently, a corresponding network of communities, with links weighted by travelers. Here, unexpectedly, the study finds that the average node degree decreased during lockdown. However, any real analysis of this community network relevant to disease spread is missing. The same is true for the obtained community distribution. Do they match political organizational units or commuting zones? Do they have typical size? The network could also serve as the basis for an agent-based or multiscale models of disease spread, which is not discussed.

We have updated this section of the analysis to provide a stronger connection between the community detection and disease spread.

We have added a discussion of the typical areal and population sizes of the detected communities in the results:

Overall, these communities were smaller than LTLAs and Travel to Work Areas (TTWAs) in terms of their size and population, although in cities like London and Manchester, communities intersected multiple TTWAs and LTLAs (Supplemental Figure 13).

And an additional supplementary figure:

**Supplemental Figure 13. Comparison of TTWAs, LTLAs, and detected communities.** Maps of 2011 TTWAs, LTLAs, and InfoMap communities detected on a specific date. While some InfoMap communities are similar in size to TTWAs and LTLAs, they do not share the same boundaries. There are fewer communities (193) than TTWAs (228) and LTLAs (380) and these communities have a smaller average area across all time periods (389.73 km^2^) compared to TTWAs (1069.14 km^2^) and LTLAs (605.28 km^2^).

We have also included further discussion of how the size and temporal stability of detected communities relates to administrative units in the discussion section:

These communities can also be used to monitor adherence to public health measures in near-real time, as changes in community structure indicate a reorganization of the patterns of travel for specific populations. We found that overall, these communities were smaller in population and area than LTLAs and TTWAs, providing a finer-grained understanding of mobility connections between areas.

And have included a discussion of how these communities are relevant for modelling of infectious disease spread, including with the use of agent-based models in the discussion section:

Community structure has been shown to influence the progression of modelled disease transmission in simulated and real-world networks (49–51). Variations in community structure can affect or limit mixing between people or between populations, giving rise to epidemics with multiple peaks or geographically-localised resurgences (52). Identifying communities is therefore important for understanding and projecting epidemic dynamics.

## Reviewer 1 - Major 4

Lastly, the authors analyze the effect of local interventions. Here the main result is that the effect of local interventions was weaker than a national lockdown. Also, the extent of communities did not always match the boundaries of local interventions in the UK. It is unclear if the authors suggest using their identified communities as boundaries for local interventions. I am missing a clear evidence that the proposed strategy of identifying communities using mobility data can improve interventions against COVID-19.

We have updated the discussion section to better discuss how the detected communities can inform responses to COVID-19 and have included a reference to a similar approach undertaken in the EU to define communities (using a different community detection method) in an effort to delimit the extent of spatially limited interventions.

The updated discussion section is as follows:

In the movement dataset used in this study, we identified groups of network nodes which have strong movement connections to each other, meaning that they share a relatively high number of travellers with other members of their community. Similar delimiting of movement communities has been applied to define “functional zones” in the European Union (53), where these zones have been recommended as a way to define the extent of locally targeted interventions when responding to spatially-limited resurgences of COVID-19.

## Reviewer 1 - Major 5

Captions of all figures are too short and do not guide the reader to quickly grasp the message of the figure. Two examples:

a. Fig1: The caption only repeats the axes labels but does not contain the message: that the number of travelers is positively correlated with new covid cases!

b. Fig2: Again, it seems to me that the message of the figure is that the percentage of Facebook users is quite homogeneous between regions and can therefore be used to say something about the full population.

Thank you for this suggestion, we agree that the figure captions were not sharing enough information with the reader. We have updated the captions of each figure in an effort to provide more information about the contents of the figure, and to help readers with their interpretation of the findings presented in the figures.

Updated figure 1 caption:

**Figure 1. Characteristics of the between-cell Facebook mobility dataset.** a) The daily average number of users included in the movement dataset. b) The probability density functions of the percentage of Facebook users in the census population for cells by country. c) The relationship between the number of Facebook users and census population for all cells. d) The spatial distribution of the percentage of Facebook users in the census population for all cells. The proportion of Facebook users to population is homogenous between regions although there are local variations in the proportion of Facebook users to population. White cells are censored due to low numbers. Note that 12 cells around the town of Swindon are missing due to a data processing error prior to data sharing.

Updated figure 2 caption:

**Figure 2. The relationship between movement and COVID-19 cases per capita.** a) Daily confirmed SARS-CoV-2 cases in each NHS region. b) The relationship between the percentage of users travelling outside their cell and the total number of confirmed SARS-CoV-2 cases per capita, by month of the study period. The rate of between-cell movement is correlated with the number of COVID-19 cases and the strength of this correlation varies through time. Dots show LTLAs coloured by their NHS region.

Updated figure 3 caption:

**Figure 3. Network structure through time.** All network connections in a) the period preceding the first national lockdown (March 10 to March 22, 2020) and b) following the first national lockdown (March 23 to April 4, 2020). The thickness of edges corresponds to their edge betweenness centrality. c) The percentage of users travelling out of their cell through time, shown as 90%, 75%, and 50% density intervals; and d) the edges that remain highly central after the introduction of national interventions. We compare the betweenness quantile for edges from March 10 to March 23, 2020 (preceding national interventions) and from March 23 to May 10, 2020 (period of national interventions). This shows a disproportionate elimination of network connections for edges which were highly central before the announcement of the first national lockdown. e) The distribution of edge betweenness and distance pre- and post-intervention on March 23rd. The red highlight demonstrates the loss of long-distance, high betweenness edges. f) The reduction of travel by distance during national interventions**.** Most long-distance edges are removed from the dataset entirely as a result of decreased travel. Note that the longest remaining connection is between Liverpool and Belfast.

Updated figure 4 caption:

**Figure 4. Community detection using the InfoMap algorithm**. Communities detected on individual days through the time series. Areas delimit the maximum extent of all cells in detected communities and are colored based on the NHS region they intersect. Network plots show the volume of travel between communities for the network of connections between communities on each day. Missing tiles which record fewer than 10 users moving have been censored for privacy and appear white. The number of communities on each day (left to right): 189, 288, 218, 209.

Updated figure 5 caption:

**Figure 5. Response to the introduction of local interventions.** a) The daily percentage of users travelling between cells in the intervention area and the connected community, and the weekly average degree of intervention cells. This shows the change in movement indicators through time, as well as the change in connectivity of this community to the remaining network of communities. b) Confirmed cases in the intervention area. c) Changes in the community structure before and after the introduction of local intervention.

## Reviewer 1 - Minor 1

Caption of fig 1. Speaks of daily reported SARS-CoV-2 tests in a) but the figure shows confirmed cases.

These are confirmed cases reported by sample date. This has been updated in the figure descriptions in the main text and supplement.

## Reviewer 1 - Minor 2

Caption of fig 2. Speaks of daily average number of users in a) but the axis label speaks about average movements. Also, areas with more than 75% of the population registered as users were removed. Why?

We removed the areas with a proportion of Facebook users to population >75% in order to better display the variance among the remaining cells. We have removed this threshold in favor of a log_10_ transformation in panels b, c, d. All cells are now included in the panels of Figure 2.

## Reviewer 1 - Minor 3

Fig 3d is absolutely unclear to me. The axis label says “number of edges”. The caption speaks about the likelihood of a journey to remain highly central in the lockdown. How do you read this?

This Figure compares the centrality of individual edges in the periods before and after the first national lockdown. We have clarified the caption of Figure 3d about the intended interpretation of this figure by the reader.

We have included the following sentences in the figure caption:

d) the edges that the likelihood of a route to remain highly central after the introduction of national interventions. We compare the betweenness quantile for edges from March 10 to March 23, 2020 (preceding national interventions) and from March 23 to May 10, 2020 (period of national interventions). This shows a disproportionate elimination of network connections for edges which were highly central before the announcement of the first national lockdown.

The intended message of this panel in the figure is that the movement network became more sparse after the first lockdown and that network edges that were eliminated after the lockdown were disproportionately those which were highly central to the pre-lockdown network.

## Reviewer 1 - Minor 4

Fig 4. Is missing labels a),b)

We have removed a) and b) labels in this figure and intend to convey the map and corresponding network plot as a component of the same figure. We have updated the figure caption accordingly, and have added a sentence to clarify the intended interpretation of the network plots which show the volume of movement between communities.

The updated figure caption is as follows:

**Figure 4. Community detection using the InfoMap algorithm**. Communities detected on individual days through the time series. Areas delimit the maximum extent of all cells in detected communities and are colored based on the NHS region they intersect. Network plots show the volume of travel between communities for the network of connections between communities on each day. Missing tiles which record fewer than 10 users moving have been censored for privacy and appear white. The number of communities on each day (left to right): 189, 288, 218, 209.

## Reviewer 1 - Minor 5

Fig 5 is also quite difficult to understand. Clarify message in the caption. Maybe zoom in into the relevant date range for the local interventions? Maybe show the number of travellers in the intervention area relative to the corresponding community, otherwise it is quite hard to see a decrease here.

We have shortened the time series displayed in the figure to better focus on the relevant time period.

**Figure 5. Response to the introduction of local interventions.** a) The daily percentage of users travelling between cells in the intervention area and the connected community, and the weekly average degree of intervention cells. This shows the change in movement indicators through time, as well as the change in connectivity of this community to the remaining network of communities. b) Confirmed cases in the intervention area. c) Changes in the community structure before and after the introduction of local intervention.

## Reviewer 1 - Minor 6

Fig S6. What is IMD?

IMD refers to the index of multiple deprivation, a measurement of relative deprivation used in the UK derived from multiple deprivation metrics. In this analysis we use multiple sources of deprivation data for each UK country (England & Wales, Scotland, Northern Ireland). We have defined IMD at the first mention in the Methods section and have ensured that this terminology is consistent through the rest of the paper.

## Reviewer 1 - Minor 7

Why is Fig S7 not in the main manuscript? It seems to be quite central as it tells us about the spatial scale of movement in the lockdown (max 50-100 km).

We agree that (previously) supplemental Figure 7 should be more prominent. We have moved this figure to a panel of Figure 3.

## Reviewer 1 - Minor 8

Same with Fig S8. It makes it clearer, which journeys are removed in the lockdown. Also, add small ticks to this plot to make the log-log axes clearer and easier to read.

We also agree that Supplemental Figure 8 would be better placed in the main text, as it contains important information about the extent of journeys after the first lockdown intervention as well as their betweenness centrality. We have also moved this figure to a panel of Figure 3.

## Reviewer 1 - Minor 9

Fig S11 is unclear to me. What should the reader see?

This figure shows the spatial alignment of communities detected using the InfoMap and Leiden algorithms. We have updated the figure caption to more clearly describe the method we used and to provide readers with an interpretation of the figure:

**Supplemental Figure 10. Comparing community detection algorithms.** A comparison of the spatial intersection between communities detected by the Infomap and Leiden algorithms through time. a) The average % overlap of InfoMap communities with Leiden communities. b) The number of Leiden communities each InfoMap community intersects, and c) the inverse comparison of Leiden communities to InfoMap communities.For a specific date, d) the maximum % areal overlap of each InfoMap community with all Leiden communities. e) The number of Leiden communities each InfoMap community intersects, and c) the inverse comparison of Leiden communities to InfoMap communities. This shows spatial alignment between InfoMap and Leiden communities, where Leiden communities tend to be larger than those detected by InfoMap.

## Reviewer 1 - Minor 10

In Fig S16, what is the dashed line? It does not seem to be a fit.

The dashed line is a line where y == x which is intended to show that the degree of cells in the network of communities is lower in the post-lockdown period compared to the pre-lockdown period (all points would fall on this line if there was no change to the network). We have updated the figure to have equal x and y limits and have added a sentence in the figure caption to describe the purpose of the line.

The updated section in the figure caption is as follows:

The dashed line indicates where the Pre-lockdown degree is equal to the Post-lockdown degree. All points would fall along this line in the event of no changes to the community network after the intervention.

## Reviewer 1 - Minor 11

The message of Figs S17-19 is also unclear. They could also benefit from zooming in the time series into the relevant time periods.

These figures benefit from the changes that have also been made to figure 5. We have zoomed the time series to the relevant period and added annotations of the relevant period for the communities shown in panels c for both figures.

**Supplemental Figure 16. Communities connected to Manchester local area restrictions.** The daily percentage of users travelling between cells in the intervention area and the connected community, and the weekly average degree of intervention cells (a). Confirmed cases in the intervention area (b). Changes in the community structure before and after the introduction of local intervention (c).

**Supplemental Figure 17. Communities connected to North East England local area restrictions.** The daily percentage of users travelling between cells in the intervention area and the connected community, and the weekly average degree of intervention cells (a). Confirmed cases in the intervention area (b). Changes in the community structure before and after the introduction of local intervention (c).

**Supplemental Figure 18. Communities connected to North West England local area restrictions.** The daily percentage of users travelling between cells in the intervention area and the connected community, and the weekly average degree of intervention cells (a). Confirmed cases in the intervention area (b). Changes in the community structure before and after the introduction of local intervention (c).

# Reviewer 2

## Reviewer 2 - Summary

The paper is well written, easy to follow and understand, even if I think that might further improve with a slightly different structure (details below). The Introduction and Methods sections provide a suitable context for the work, but some relevant references are missing (details below). The figures are high quality and appropriate for illustrating the ideas and work presented. Overall, the analysis is sound, the paper is self-contained and presents a suitable selection of results to support the main findings.

While I think the paper is interesting and has merit, it mainly reports a descriptive data analysis, therefore it will greatly improve if the authors will widen the discussion and implications of their findings in the context of public health interventions.

We thank the reviewer for these positive comments and agree that the paper would improve by expanding the discussion of our findings. We have expanded the discussion and description of implications including the useful references provided by the reviewer:

We identified geographically distinct communities with strong interconnections that are relevant to policy responses focused on limiting transmission in response to geographically-limited disease resurgences. These communities delimit the boundaries of areas with strong internal movement connections and therefore provide empirical boundaries that can inform policy responses or form the basis for spatially-explicit transmission models. Community structure has been shown to influence the progression of modelled disease transmission in simulated and real-world networks (49–51). Variations in community structure can affect or limit mixing between people or between populations, giving rise to epidemics with multiple peaks or geographically-localised resurgences (52). Identifying communities is therefore important for understanding and projecting epidemic dynamics.

There is a demonstrated relationship between human mobility and transmission of COVID-19 relating both to the spatial introduction of disease, where connectivity is correlated with the time of first introduction (11), and rates of movement in specific locations, which are correlated with disease transmission (13). In the movement dataset used in this study, we identified groups of network nodes which have strong movement connections to each other, meaning that they share a relatively high number of travellers with other members of their community. Similar delimiting of movement communities has been applied to define “functional zones” in the European Union (53), where these zones have been recommended as a way to define the extent of locally targeted interventions when responding to spatially-limited resurgences of COVID-19.

## Reviewer 2 - Major 1

Relationship between movement and COVID-19 cases: corroborated by the correlations shown in Figure 1, the authors suggest that there is an “intuitive relationship between increased rates of travel outside of local areas and increased COVID-19 prevalence”. This statement needs better framing and a deeper discussion. First, the volume of travels outside a local area would be relevant for the local spreading only under the hypothesis that such travels are mainly due to commuting patterns that come back to the local area of origin. While this might be true for the data used in this paper, some discussion about this point is needed. Secondly, it is well known that -in the context of infectious diseases spreading-, travel fluxes are associated with the arrival time of the disease in not-yet-affected areas [Brockmann2013, Balcan2009]. However, after the initial seeding the local outbreak unfolds with an exponential growth and thus further importation of cases should not play a major role in shaping the epidemic curve. Therefore, the author should elaborate on this concept to let the reader grasp their intuition, and why such result is interesting. Finally, I think that a normalized quantity for the number of cases (as reported in Supplemental Figure 3) would be a more suitable observable, as the raw count of cases is clearly influenced by the number of people living in certain area.

We agree with the reviewer, and have made an effort to improve the paper with a more detailed discussion and analysis of the way that the human movement patterns we observe are related to dynamics of disease transmission. We have updated the introduction and discussion sections to discuss the way the human movement is related to initial seeding of disease along movement networks, but also the way that rates of movement are used as a proxy for social contact and have been shown to correlate with disease transmission.

Additions to the introduction section:

The spread of COVID-19 through travel networks has been demonstrated in China, where connectivity to Wuhan was shown to predict the timing of arrival of COVID-19 cases in each region [(11,12)](https://www.zotero.org/google-docs/?broken=OxS6nU). The volume of mobility has also been shown to correlate with transmission of COVID-19, where movement can be used as a proxy for the measurement of the degree of social distancing [(13)](https://www.zotero.org/google-docs/?broken=Ufv81B).

Additions to the discussion section:

There is a demonstrated relationship between human mobility and transmission of COVID-19 relating both to the spatial introduction of disease, where connectivity is correlated with the time of first introduction (11), and rates of movement in specific locations, which are correlated with disease transmission (13). In the movement dataset used in this study, we identified groups of network nodes which have strong movement connections to each other, meaning that they share a relatively high number of travellers with other members of their community. Similar delimiting of movement communities has been applied to define “functional zones” in the European Union (53), where these zones have been recommended as a way to define the extent of locally targeted interventions when responding to spatially-limited resurgences of COVID-19.

We have also provided a more detailed explanation of the structure of the movement dataset and our use of “movement between cells” as an indicator of the volume of travel in a specific location. We have made the assumption that individuals leaving a cell primarily return to the same cell in a given period explicitly and attempted to explain how the structure of the dataset limits the ability to identify people travelling in a local area or simply remaining in one location (i.e. home). We have updated the methods section in an effort to clarify this:

We use movement outside of local areas as a measurement of movement from one cell to any other cell in the network. The movement data also records connections from one cell to the same cell in sequential periods. This type of movement may indicate movement within a local area (less than the area of a given cell), or completely stationary individuals. Because of the spatial resolution of the data, it is not possible to quantify the volume of within cell movement that corresponds to each of these behaviours within the same cell. We therefore use the volume of movement outside of the cell as a measurement of the overall movement behaviour, making the assumption that travellers leaving a cell will primarily return to the same cell in sequential periods.

We agree that using confirmed cases normalised by population is a more appropriate way to assess the correlation between rates of movement and disease incidence. We have moved the figure, previously Supplementary Figure 3, to Figure 3 and vice versa.

## Reviewer 2 - Major 2

Characteristics of the between-cell Facebook mobility dataset: the authors considered “the percentage of Facebook users per cell […] homogeneous across the study area (Figure 2d)”. However, Figure 2B shows distributions with an average value ~10% and long tails up to ~70%, that seems quite heterogeneous. The authors should clarify or better frame this point. Moreover, the correlation reported in Figure 2C would definitely benefits from a log-log transformation, to

limit the impact of few outlying data points in skewing the estimate of the correlation coefficient.

We have updated Figure 2 to add a log transformation to panels (c) and (d) and agree that this provides a clearer demonstration of the relationship between the number of facebook users and census population. We have also removed the threshold for cells with >75% Facebook users to population. We originally used this threshold to highlight differences in the majority of cells, but achieve the same effect using a log transformation. These values are outliers but should be included in the main figure.

We have also updated the results to specify that, while there is variation in the proportion of Facebook users to population, we do not observe strong association between this proportion and any of the demographic variables we test. This is different from spatial homogeneity and have rewritten this discussion in the results:

The percentage of Facebook users per grid cell was comparable in the four nations of the UK (Figure 1b). The population of Facebook users was highly correlated (R = 0.97, p < 2.2e-16) with census population estimates for all cells (Figure 1c), although there is variance in the proportion of Facebook users to census population across cells (Figure 1d). We compared the proportion of Facebook users to identify whether the distribution of Facebook users was biased in relation to a specific demographic variable, finding no strong associations between the percentage of Facebook users and the average age, percent minority ethnic, population density, or IMD of each cell (Supplemental Figures 3-5).

## Reviewer 2 - Major 3

Network Structure: the authors compared the betweenness quantile for links of the mobility networks extracted before the national interventions (from March 10 to March 23) and during the national interventions (from March 23 to May 10). My first concern is that the two networks are not comparable as they are built aggregating 13 days and 47 days respectively. Therefore, I would expect some major differences even in the absence of exogenous interventions. In principle, a longer aggregation window might result in a denser network (light links from rare travels are reinforced with longer observation time). The authors might consider to perform the analysis on mobility networks extracted on comparable time windows. Moreover, the community detection analysis seems to be performed on daily mobility networks: relying on the same temporal aggregation would help the reader compare the findings of the different network analysis.

We agree that it is more appropriate to compare periods of equal length and have updated the analysis to compare centrality of connections in equal length periods.

As a side note: it is interesting that in the previous version, although the second period of aggregation is longer, and would therefore be expected to be denser simply because of the longer period of observation, the opposite is true, as the second network is more sparse than the first, particularly for highly central journeys. Nonetheless, the analysis is improved by the comparison of equal time periods which is present in the updated version of Figure 3.

## Reviewer 2 - Major 4

Since the mobility network is largely destroyed by the national interventions, besides the information on betweenness quintiles, some coarser information about mobility network resilience could be helpful to better understand how the system evolved (e.g. how many links of the pre-intervention network are preserved in the post-intervention period? How many new links were created, if any?).

We agree that this section would be improved by sharing general metrics that describe the difference between the two networks. We have included a summary of your recommended measurements, as well as a few other whole-network metrics. We have also included a discussion of the changes in the distance of connections, something that we mentioned previously, but for which we did not provide an explicit analysis.

We have updated the results section accordingly:

Overall, the network was reduced by 10,690 edges, with only 46 new edges observed in the post-intervention period. All of these newly created edges had a distance less than 47km. The network also became more disconnected, with the number of components increasing from 23 (largest component size: 3394) to 37 (largest component size: 2579).

## Reviewer 2 - Major 5

Some communities detected on mobility networks of individual days are quite stable across time. A natural question is to verify if such pattern persists also when mobility data are aggregated at different temporal scales (e.g. weekly, monthly,…).

We have conducted the suggested analysis and found that the communities are stable when aggregated by day, week or month. We make this comparison using the Normalised Mutual Information measure of community labels [Danon et al. (2005)].

We have added the new Supplementary Figure 17:

**Supplemental Figure 17. Community detection with different time aggregations.** Normalised Mutual Information, a measure of similarity between community partitions, measured for InfoMap communities detected in networks aggregated to daily, weekly, and monthly periods. The partition of each network is compared to its parent time aggregation (days to overlapping weeks, weeks to overlapping months).

and reference this in Results with the following:

We found strong stability of communities calculated for networks aggregated to daily, weekly, and monthly intervals, with a Normalised Mutual Information, a measure of similarity between network partitions, between 0.89 and 0.92 for all partitions (Supplemental Figure 17).

## Reviewer 2 - Major 6

The authors show how the community structure in four areas (Leicester, Manchester, the North West, and the North East) seems to be stable before and after some local interventions, with the only exceptions of some grid cells. There has been some recent work in assessing the association between socio-economic status and change of mobility induced by COVID-19. I am aware that it is rather uncouth of me to suggest referencing my own work, but our paper [www.medrxiv.org/content/10.1101/2020.11.16.20232413v1](http://protect-eu.mimecast.com/s/fZzRCmy54upNjKviGw5eN?domain=medrxiv.org) seems particularly appropriate here as it explore such phenomenon at the microscale of city neighborhoods. I would not expect the authors to implement an extensive analysis in the current paper, but it would be great if they could add some insights about deprivation levels of the cells that change community membership and in general it should certainly be considered as further work. The authors could also consider to widen their discussion to include some missing reference in this direction, such as [Jay2020, Pullano2020, Gauvin2020]. Overall, the authors might consider elaborating a bit on this point, as the only analysis performed is in Supplemental Figure 6 and poorly commented in the main paper. I believe that this aspect could be important to strengthen the paper, as the implication of such analysis might suggest that deprived areas respond differently under top-down intervention, thus potentially limiting their effectiveness on more deprived and vulnerable population.

We thank the reviewer for raising this important point and agree that the role of socioeconomic status and mobility is critical, both to disease transmission and to adherence to interventions. We have read the reviewer’s work and others referenced. However, the very fine scale data used there are not available in the Facebook data, which are provided only as fixed size Bing Tiles of approximately 4.6km^2^. This is especially poor resolution within cities, where there can be very local changes in socioeconomic status and therefore effect on mobility. We did explore socioeconomic status extensively prior to this manuscript, but concluded it was unfortunately not possible with these data. This granularity limits the types of analysis these data can be used for, and we hope to use finer-grained data in the future to better explore this question in the UK, as socioeconomic status has been shown to be important in Italy, the USA, and other countries. In the present publication, this figure is intended to show similarities in the movement patterns of across IMD quintiles.

We have also extended the introduction to discuss this application, and have included the suggested references:

During the COVID-19 pandemic, mobility data has been used to assess adherence to movement restrictions (13,14), the impact of movement restrictions on the transmission dynamics of COVID-19 (15–17), and demonstrate differential adherence to movement restrictions among socioeconomic groups (18–21).

**Reviewer 2 - Major 7**

The authors might be interested in reading (and probably citing) some references closely related to this work [Iacus2020, Bonato2020]. In those papers, the authors investigated the time evolution of data-driven Mobility Functional Areas (geographic zones with high degree of intra-mobility exchanges) before and after national interventions.

Thank you for these helpful resources, particularly Iacus et al. 2020, who use the walktrap algorithm to cluster provinces based on their mobility connections. We have updated the discussion section to include this reference, and included a discussion of the ways that community detection algorithms are already used to identify “functional zones” (urban, or otherwise), and their recommendation as the basis for spatially targeted interventions. The updated section of the discussion is as follows:

Similar delimiting of movement communities has been applied to define “functional zones” in the European Union (53), where these zones have been recommended as a way to define the extent of locally targeted interventions when responding to spatially-limited resurgences of COVID-19.

## Reviewer 2 - Minor 1

For the sake of readability, I suggest to move Figure 2 and the considerations about the representativeness of the dataset before Figure 1, where such data are used.

We agree that it is clearer to have the analysis of cases and movement rates follow the discussion of the representativeness of the data. We have rearranged these sections as suggested and swapped Figures 1 and 2. The rearrangement is not shown in tracked changes (Google Docs issue).

## Reviewer 2 - Minor 2

Figure 3C: an explanation about the meaning of the different colors of the shaded areas is missing.

These are density intervals (90%, 75%, 25%). We have updated the caption of Figure 3c to include this information as follows:

*The percentage of users travelling out of their cell through time, shown as 90%, 75%, and 50% density intervals.*

## Reviewer 2 - Minor 3

In the first paragraph of page 8, the authors refer to Supplemental Figure 9 as a comparison of Infomap vs. Leiden communities, but they should probably refer to Supplemental Figures 10 and 11.

Thank you, we have updated the references to the supplemental figures to ensure that we reference these figures comparing the Infomap and Leiden algorithms.

## Reviewer 2 - Supporting Research

Brockmann, D. and Helbing, D., 2013. The hidden geometry of complex, network-driven contagion phenomena. science, 342(6164), pp.1337-1342.

Balcan, D., Colizza, V., Gonçalves, B., Hu, H., Ramasco, J.J. and Vespignani, A., 2009. Multiscale mobility networks and the spatial spreading of infectious diseases. Proceedings of the National Academy of Sciences, 106(51), pp.21484-21489.

Jay, J., Bor, J., Nsoesie, E.O., Lipson, S.K., Jones, D.K., Galea, S. and Raifman, J., 2020. Neighbourhood income and physical distancing during the COVID-19 pandemic in the United States. Nature human behaviour, 4(12), pp.1294-1302.

Pullano, G., Valdano, E., Scarpa, N., Rubrichi, S. and Colizza, V., 2020. Evaluating the effect of demographic factors, socioeconomic factors, and risk aversion on mobility during the COVID-19 epidemic in France under lockdown: a population-based study. The Lancet Digital Health, 2(12), pp.e638-e649.

Gauvin, L., Bajardi, P., Pepe, E., Lake, B., Privitera, F. and Tizzoni, M., 2020. Socioeconomic determinants of mobility responses during the first wave of COVID-19 in Italy: from provinces to neighbourhoods. medRxiv.

Iacus, S., Santamaria, C., Sermi, F., Spyratos, S., Tarchi, D. and Vespe, M., Mapping Mobility Functional Areas (MFA) using Mobile Positioning Data to Inform COVID-19 Policies, EUR 30291 EN, Publications Office of the European Union, Luxembourg, 2020, ISBN 978-92-76-20429-9, doi:10.2760/076318, JRC121299.

Bonato, P., Cintia, P., Fabbri, F., Fadda, D., Giannotti, F., Lopalco, P.L., Mazzilli, S., Nanni, M., Pappalardo, L., Pedreschi, D. and Penone, F., 2020. Mobile phone data analytics against the covid-19 epidemics in italy: flow diversity and local job markets during the national lockdown. arXiv preprint arXiv:2004.11278.

# Reviewer 3

## Reviewer 3 - Summary

This was a great read. It was very interesting to see fine-scaled movement data quantified and associated in this way. I think the content stands on its own, but could be made more compelling by a stronger connection between movement data and virus reproduction rates.

I find myself wondering about the relationship between cell-to-cell travel and transmissible contact rates. Is it possible to characterize the types or proportion of trips that are captured by these movement data (for the average/median individual)? Commutes to work or school, trips to the grocery, etc. For instance there might be statistics on commuting that would allow you to estimate the proportion of commutes that leave a level-13 cell.

We thank the reviewer for these positive comments. We have more clearly defined the important link between mobility data and their relevance for disease transmission by including further references, clarifying text in the introduction, results, and discussion, and updating the results section of our analysis dealing with the correlation between rates of movement and confirmed COVID-19 cases.

We agree that it would be very interesting to further investigate the way that individual patterns are represented in these movement data. However, Facebook data are limited by 3 major privacy-oriented features: 1) The spatial resolution of the data is coarse in cities, where most commuting happens. 2) journeys that intersect time periods (though not for movement through individual cells) are censored, which is especially important for school, where travel around 8am and 4pm is critical, and 3) we have no information about the specific characteristics of Facebook users in the dataset which could help in estimating individual or group-specific patterns of movement. We have extended the discussion on limitations of these data in the methods and discussion sections.

## Reviewer 3 - Major 1

Facebook Data: From the text it is difficult to determine if the movement data is (1) simply a geolocation taken every 8 hours, (2) change of map cell at any time during each 8 hour period, or (3) something else. If it is (2), can an individual have multiple journeys in a single 8 hour period?

The structure of the movement data is somewhat different from (1) and (2), as it is an aggregation of multiple locations taken during sequential 8-hour periods. We have updated the methods section to provide more clarity about the specific structure of the movement data:

We used movement data, describing users’ modal locations in map cells in sequential 8 hour periods. This means that, in each period, a user is assigned to the cell in which they record the largest number of locations. For this individual, the beginning and end points of a journey are defined as their location in sequential periods. This individual’s movement is then aggregated with others to form a weighted Origin-Destination matrix of movement between grid cells.

## Reviewer 3 - Major 2

Demographic information: The first sentence is accurate, but a lot to unpack. I had to read through this section, look at fig S5 and then go back and reread the first sentence to fully understand it. I would suggest leading with “to identify relationships between the percentage of Facebook users and demographic factors, we…” or somesuch. Then describe the details. This is only a readability suggestion, I respect the authors’ preference.

Thank you for this suggestion, we have updated the section of the results where the relationship between the proportion of Facebook users and census population is compared to demographic variables to include a sentence that describes the motivation for this analysis. It now reads:

To understand the representativeness of Facebook data to the general population, we explored the size of the population of Facebook users included in the movement dataset, and compared this population to 2019 UK census population estimates.

## Reviewer 3 - Major 3

Community Detection: What is the cost/objective function? Is there a penalty in the algorithms for adding additional communities?

S10 Fig: If it is relatively easy, it would be interesting to draw the outline of Leiden communities over the InfoMap communities.

The Leiden algorithm tries to maximize the modularity of communities while the Infomap algorithm tries to minimize the description length of a random walker along the network. We have expanded the description of both algorithms in the methods section to provide more detail to readers about the specifics of each algorithm. The modified text now reads:

InfoMap is a community detection algorithm based on the Map equation which records the movements of a random walker along the network. The algorithm tests network partitions, attempting to identify the partition that minimizes the description length required to describe the walker’s path (31). In other words, the algorithm compresses the description of movements in parts of the network where the walker spends a large amount of time, thereby forming communities from these strongly connected sections of the network. The Leiden algorithm maximizes the modularity of different node partitions. To do this, the algorithm assigns network nodes to a partition, assesses the modularity of the partitioned groups, aggregates the nodes in a given partition, and repeats this process until there is no further improvement in the modularity of the partitions. This process results in a partition in which communities possess stronger connections to members of their own community than to other network nodes (32,33). There is a penalty for both algorithms if they identify a number of communities larger than the minimum number required to maximize their respective objective functions (InfoMap: description length, Leiden: modularity).

We have also included the suggested supplementary figure for a selection of dates:

**Supplemental Figure 9. InfoMap and Leiden community detection methods.** A comparison of communities detected with the InfoMap (red) and Leiden (black) methods for a selection of dates.

## Reviewer 3 - Major 4

In addition to using these methods for geographic definition of interventions and an indicator of higher transmission, I would consider discussing how these metrics could be used as real-time feedback concerning the effectiveness of intervention mandates.

This is a valuable suggestion and we have updated the discussion section to include this use of the detected communities. Because these communities reflect the aggregated patterns of movement for the population of a specific location, the stability or change in community structure would be useful for assessing the patterns of movement around spatially targeted intervention areas. We have updated the discussion section accordingly:

These communities can also be used to monitor adherence to public health measures in near-real time, as changes in community structure indicate a reorganization of the patterns of travel for specific populations.

## Reviewer 3 - Major 5

A stronger connection between transmission and movement data would be compelling. It would be interesting to compare the movement data to effective reproduction number estimates and check for temporal correlation. This would remove transmission lags and ‘ramp-up’ from the comparison to cases and potentially make for a more direct connection to transmission dynamics. Is this something you considered?

We thank the reviewer for this suggestion, which is something that we tried during the analysis. Unfortunately, we could not align the spatial reference of available *R_t_* estimates or newly-calculated *R_t_*s from case data with the geographic extent of our newly-identified movement communities. Publicly-available *R_t_* estimates were at NHS region level [Epiforecasts, 2021] (of which there are 7 in England), which is a much coarser spatial resolution than our InfoMap communities (approximately 200). We recalculated *R_t_* estimates for LTLAs (of which there are 326 in England) for which daily case data were available, but the geographic boundaries of LTLAs are misaligned with our mobility communities: some are larger and some smaller than the identified communities. We also attempted to use publicly-available MSOA-level data to re-estimate *R_t_* (there are 6791 MSOAs in England), but MSOA case data are available as a 7-day rolling mean which over-smooths the *R_t_* estimates, making those inappropriate for comparison to the mobility data. We agree that this would be an interesting area for future analysis but is not currently possible with available data.

We have added further discussion of the relationship between movement and disease transmission in networks with community structure in the discussion section in order to clarify the relationship between detected communities and disease transmission.

[Epiforecasts, 2021] <https://epiforecasts.io/covid/posts/national/united-kingdom/>

## Reviewer 3 - Minor 1

Perhaps remove “, and network service providers” from the last sentence of first paragraph. Repeated ‘and’ and ‘network providers’.

We have updated this sentence in the manuscript.

## Reviewer 3 - Minor 2

ONS data citation?

We have used population and demographic data from the Office for National Statistics (England), Northern Ireland Statistics and Research Agency, Scottish Government, and Welsh Government. We have included references for these agencies and cited the specific datasets we use in the methods section.

## Reviewer 3 - Minor 3

S13 Fig b and c: are these ordered row-wise or column-wise?

We have updated the figure caption to describe the temporal pattern for each panel.

## Reviewer 3 - Minor 4

COVID-19 Data: I do not believe LTLA is previously defined

We have defined LTLAs (Lower-tier local authorities) at the first mention in the methods section and removed the redefinition of this acronym in the results section.

## Reviewer 3 - Minor 5

Facebook Data: is there any latency or is the data for a given day released the following day?

The data is released approximately 48 hours delayed. We have included this in the methods section:

*This data is generated from the population of Facebook users with location services actively enabled and is released approximately 48 hours after data collection.*

**Reviewer 3 - Minor 6**

S2 Fig caption: are these level 12 or 13 cells? For what administrative boundary? I assume for all of the U.K., but please clarify.

We have specified that Supplemental Figure 2 shows the tiles included in the mobility dataset which are aggregated to level 12 tiles.

The updated figure caption is as follows:

**Supplemental Figure 2. The number of cells included in the mobility dataset (zoom level 12).** Cells recording fewer than 10 persons moving between cells along any connection are censored from the dataset to preserve user privacy.

# Editorial Notes

**Have the authors made all data and (if applicable) computational code underlying the findings in their manuscript fully available?**

The [PLOS Data policy](https://protect-eu.mimecast.com/s/0attCnO14cXN7qniJJkdH?domain=journals.plos.org) requires authors to make all data and code underlying the findings described in their manuscript fully available without restriction, with rare exception (please refer to the Data Availability Statement in the manuscript PDF file). The data and code should be provided as part of the manuscript or its supporting information, or deposited to a public repository. For example, in addition to summary statistics, the data points behind means, medians and variance measures should be available. If there are restrictions on publicly sharing data or code —e.g. participant privacy or use of data from a third party—those must be specified.

Reviewer #1: **No:** Data used in this study are available from the Facebook Data for Good Partner

Program by application. The computational code is not available.

Reviewer #2: **No:** Data used in this study are available from the Facebook Data for Good Partner Program by application.

Reviewer #3: Yes

**Figure Files:**

While revising your submission, please upload your figure files to the Preflight Analysis and Conversion Engine (PACE) digital diagnostic tool, [https://pacev2.apexcovantage.com](https://protect-eu.mimecast.com/s/0lAsCqZ14t1j8P9TN6TSY?domain=pacev2.apexcovantage.com). PACE helps ensure that figures meet PLOS requirements. To use PACE, you must first register as a user. Then, login and navigate to the UPLOAD tab, where you will find detailed instructions on how to use the tool. If you encounter any issues or have any questions when using PACE, please email us at [figures@plos.or](mailto:figures@plos.org)g.

**Data Requirements:**

Please note that, as a condition of publication, PLOS' data policy requires that you make available all data used to draw the conclusions outlined in your manuscript. Data must be deposited in an appropriate repository, included within the body of the manuscript, or uploaded as supporting information. This includes all numerical values that were used to generate graphs, histograms etc.. For an example in PLOS Biology see here: [http://www.plosbiology.org/article/info:doi/10.1371/journal.pbio.1001908#s5](http://protect-eu.mimecast.com/s/w36HCr914Srl80xcNjbyP?domain=plosbiology.org).

**Reproducibility:**

To enhance the reproducibility of your results, we recommend that you deposit your laboratory protocols in protocols.io, where a protocol can be assigned its own identifier (DOI) such that it can be cited independently in the future. Additionally, PLOS ONE offers an option to publish peer-reviewed clinical study protocols. Read more information on sharing protocols at [https://plos.org/protocols?utm_medium=editorial-email&utm_source=authorletters&utm_campaign=protocols](https://protect-eu.mimecast.com/s/aD76CvZ14tEz7jvsyDjQt?domain=plos.org)
